# Benefits of crowd-sourced GPS information for modelling the recreation ecosystem service

**DOI:** 10.1371/journal.pone.0202645

**Published:** 2018-10-15

**Authors:** Coline Byczek, Pierre-Yves Longaretti, Julien Renaud, Sandra Lavorel

**Affiliations:** 1 Laboratoire d’Ecologie Alpine, CNRS—Université Grenoble Alpes, CS, Grenoble, France; 2 Université Grenoble Alpes, CNRS, Inria, Grenoble INPINP Institute of Engineering Univ. Grenoble Alpes, LJK, Grenoble, France; 3 Université Grenoble Alpes, CNRS-INSU, IPAG, CS, Grenoble, France; Beijing University of Posts and Telecommunications, CHINA

## Abstract

Modelling cultural ecosystem services is an enduring challenge, raising issues about the integration and spatialization of immaterial values and benefits, and their contingency on local preferences. Building on the Recreation Opportunity Spectrum framework, we present a novel methodology for assessing the recreation service using GPS tracks downloaded from crowd-sourced websites: the Grelou model (Georeferencing REcreation in Local OUtdoors), here applied to the Grenoble living area (French Alps). GPS tracks revealed the complete spatial extent of visitor presence and enabled modelling visitation networks for ten recreation activities with great spatial accuracy, thus providing a spatial estimate of recreational multifunctionality–expressed as the sum of networks. After coupling track networks with landscape preference and proximity factors, Grelou assessed the recreation service as a combination of opportunity and preferences, and identified recreation hotspots of different profiles such as aroundoor leisure or outdoor sport. We performed an online survey among local sports associations using an interactive map to select districts visited by respondents (~1000 people). The declared visitor presence for recreation purposes was highly spatially congruent with Grelou outputs (R^2^ = 0.89). Detailed analysis of responses on selection criteria for recreationists validates our choice of critical factors underlying both the recreation opportunity potential and the expected visitation frequency over the whole study area. We also analyzed outputs of the InVESt recreation model against the same visitation explanatory factors. Differences between the two models allowed us to pinpoint biases and weaknesses in the InVESt recreation modelling framework based on crowd-sourced photographs. By making use of an increasingly available data source (GPS tracks), Grelou offers a standardized and flexible way to assess the recreation service associated with multiple recreation practices. Its high spatial accuracy supports the analysis of spatial relationships with other ecoystems services and the integration of recreation into environmental assessments, land management and planning.

## 1. Introduction

After a decade of conceptual and methodological development, current ecosystem service research faces the challenge of meeting its ambitions and making the concept operational for informing policy and decision-making [1]. While the consideration of cultural services holds a strong potential for enhancing the sustainable management of landscapes [2], their quantification remains a critical challenge, as these services strongly vary according to people’s perceptions as well as to biophysical features [3,4]. In western developed contexts, the last two decades have seen an unprecedented massification of outdoor recreation, along with diversification and hybridization of activities. This generates both an increased demand and diversification of supply of recreation opportunities, with a resulting increased need for their quantification to support management and policy.

Based on a synthesis of published studies, [5] identified four categories of variables commonly used for quantifying and subsequently mapping recreation as a service: land use, accessibility, valuation and landscape aesthetics. The fact that the latter two sets of variables are strongly influenced by individual perceptions constitutes an enduring stumbling block for modelling recreation services, and indeed cultural ecosystem services in general due to strong site- and social group specificities. Therefore many studies of the recreation ecosystem service (‘recreation ES’ henceforth) have relied on surveys to assess users preferences and uses [6,7]. Other methods have been developed in order to assess recreation ES without resorting to surveys, and thus support decision-making at a low cost and with little data and processing requirements. These methods are generic, contrary to survey-based assessments which are site- or region-specific. As an example, Schirpke and colleagues recently assessed recreation supply potential, demand and flow in the European Alps using respectively indicators of accessibility, resident and tourist density and photographs on social media [8]. Generic methods use models combining attributes describing land use, accessibility and landscape aesthetic value of varying complexity, with this complexity usually increasing from broader, e.g. continental scale, to small regions or landscapes [5]. Conversely, their robustness is difficult to validate without surveys at specific sites.

The range of recreational opportunities offered by an area, a concept explored in recreation ecology research as the Recreation Opportunity Spectrum (ROS) [9], can be assessed as the combinations of varying levels of independent factors representing for instance the capacity of ecosystems to provide recreation ecosystem services–or Recreation Potential–and accessibility which enables populations to experience outdoor recreation in these ecosystems [8]. The Recreation Potential can be estimated using case-relevant sets of spatial indicators which are assumed to be positively correlated with ecosystem attractiveness [10–12]. Accessibility accounts for proximity of recreation sites and for availability of recreation facilities such as hiking trails [13]. ROS models of recreation ES provide useful information for the management of recreation areas and for land planning at different scales [5,10,13–15] but need to be validated against survey data to account for actual visitation rates.

Alternative methods for modelling recreation ES based on actual visitation have emerged recently based on the increasing availability of social media data [16,17]. These methods use geo-referenced pictures available on crowd-sourced web sites (e.g. FlickR^®^ for the InVEST recreation module [17,18], or Panoramio^®^ [16]) to estimate visitation rates as a proxy for recreation value. Tenerelli et al. [15] demonstrated their ability to capture spatial patterns of public preferences depending on landscape settings defined as physical and built characteristics. However, using pictures as input data for spatial regression attests for visitor presence but may present some bias when the subject of the pictures cannot be filtered and may include non-nature-related content. Another problem lies in the spatial discrepancy inherent to repositories of georeferenced pictures which indicate the position of the photographer and not of the subject which is located at an unknown distance. Also, the spatial nature of pictures (points) captures visual hotspots mainly, but not all areas of visitor presence. Finally, visitor presence and propensity to report visits on social media are notoriously biased socially [11, 19].

The spatially-explicit modelling of recreation ES therefore still remains a challenge requiring novel methods [20]. This study aimed to build on the complementary strengths of both the Recreation Opportunity Spectrum and crowd-sourced models, to develop a novel model of the recreation ES applied to the Grenoble urban area, in the French Alps. As in many similar regions, recreation activities have over the last three decades shifted from longer and effort-demanding traditional practices such as hiking, cross-country skiing or mountaineering, to more playful and less-than-a-day trips [21]. In densely populated European mountain regions, demand for recreation services is thus increasing rapidly, mostly in nearby areas [5,8,21]. Sites and itineraries are no longer concentrated around mountain resorts but show increasingly intense and spatially diffuse visitation [22]. Impacts on the environment and conflicts with other users and among recreationists increase as a result [22,23].

As examples of previous applications of the ROS, in the Netherlands a national-level model of spatially diffuse recreation activities, estimated Recreation Potential through preferences of Dutch people for ecosystem types, and the availability of cycling roads [10]. At the European scale, Paracchini et al. [12] assessed the opportunities for short-distance daily trips for ordinary recreation, regardless of the activity type. In the Grenoble region as in most parts of the European Alps, sports and mountains are strong motivations for inhabitants. Hardly any single sport dominates activities, and individuals rather tend to cumulate activities, with a marked preference for modern, technical and hybrid sports, as well as performance-oriented, off-track and sometimes even life-exposing practices. Specifications for a useful tool for supporting management of multi-activity areas such as European or North American mountain regions would require providing information on the spatial extent of each activity. This is a challenging task since all activities use road and trail networks, but little information is available as to where exactly and to which extent. Such a methodology should also be standardized so that the modelling effort would not increase dramatically with the number of included activities.

Lastly, this increased recreational use of nature has concurred with the emergence of social media which support virtual communities of users sharing real time information on itineraries and conditions. In particular, many outdoor recreationists record their itineraries with GPS devices which they later post on dedicated websites for experience-sharing and performance comparison.

Building on current state-of-the art for modelling the recreation ES, we address the challenge of accounting for recent transformations of practices by enhancing the ROS model through the use of publically-available georeferenced data to refine the accessibility factors associated with recreation facilities. We developed a standardized method based on the use of GPS tracks to model the spatial extents of itinerary-based recreational activities. This way, we built for each activity a ‘recreation opportunity network’, representing the fraction of the road and trail networks which are used by recreationists for the practice of a particular activity, as well as a partial estimation of the extent of off-track practices.

In this paper, we describe this innovative modelling framework and present results for the Grenoble urban region, emphasizing hot and cold spots of recreation activities and their determinants. We then compare predicted recreational values with outputs from InVEST and validate results with an online survey. We discuss the relevance of our modelling framework for management and decision-making in a context of increasing demand for multiple recreational activities.

## 2. Model description

### 2.1 Study site

**Fig 1:**
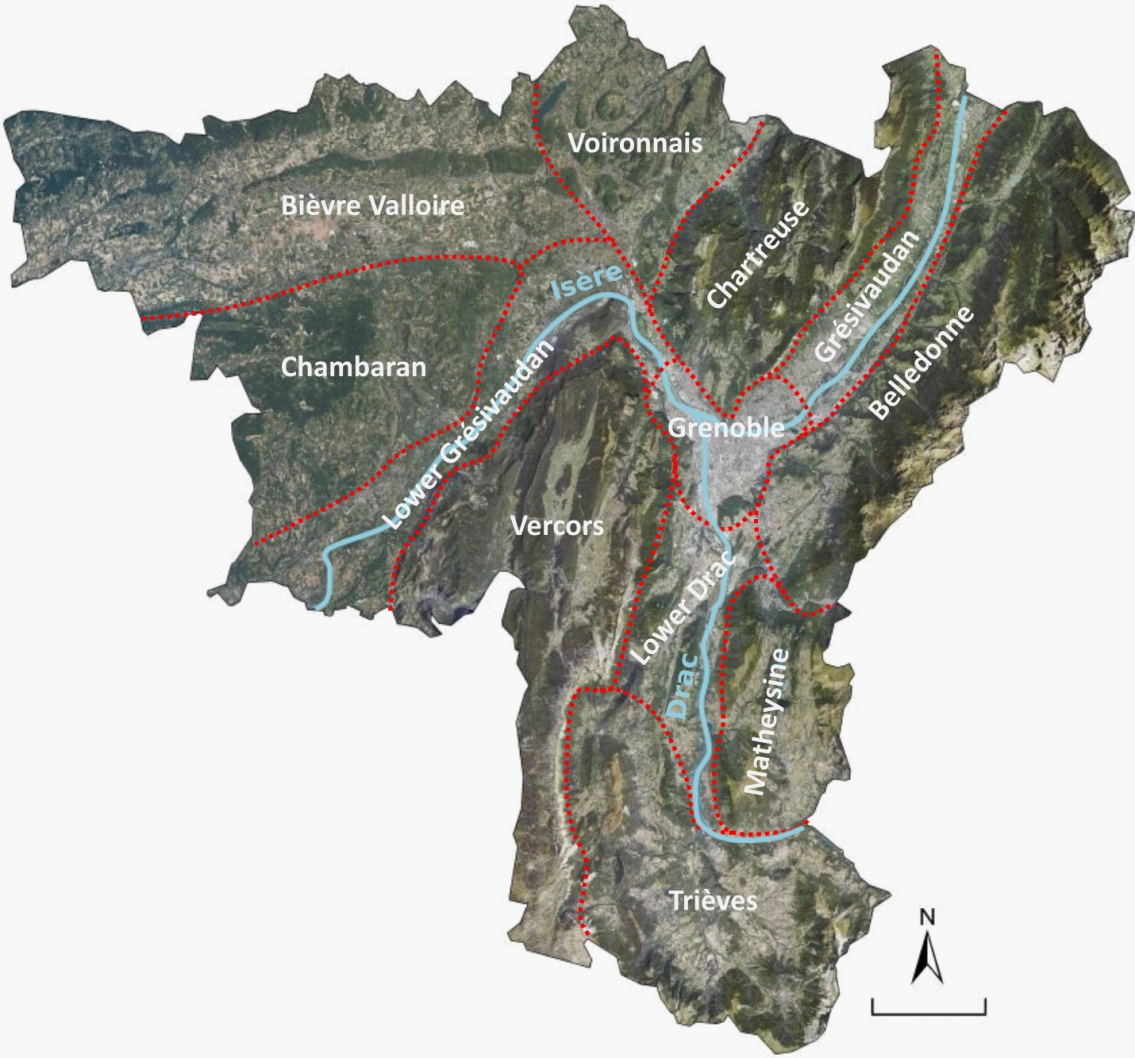
Overview of the Grenoble urban area and its main districts. Map background: IGN orthophotography.

The project study area encompasses the working and living area of the Grenoble city with a diversity of natural and human landscapes over 4450km^2^ (Fig 1). The area comprises three major mountain ranges–Belledonne, Chartreuse and Vercors–and two other hilly areas–the Matheysine and Trièves plateaus. These are separated and connected by three U-shaped valleys: the Lower and Median Grésivaudan and the Drac valley, which intersect in Grenoble. Land cover was mapped at 15 m resolution combining a multi-source merging method and aerial photo-interpretation (Grenoble Land Cover map henceforth [24]). Mountain ranges are predominantly covered by forests (31% coniferous, 20% deciduous, 14% mixed) and alpine grasslands (15%), with only 4% built-up land. In the plains which host most of the population 17% of the area is built-up, surrounded by agricultural landscapes (24% crops, 19% grassland) with more restricted forested areas (28%, broadleaved forests only).

Around 730,000 inhabitants live in the Grenoble region, of which 440,000 are concentrated in the Grenoble urban area. The remaining population lives mostly in the agricultural valleys. Mid-size towns are scattered across the agricultural plains and hills of the Bièvre-Valloire territory, as well as in the valleys within the Chartreuse and the Vercors and on Belledonne foothills.

Besides the regular forestry and agricultural activities, the mountain ranges are heavily visited by the young, dynamic and wealthy urban population for recreation. Two regional parks in Chartreuse and Vercors protect these remarkable mountain landscapes, and another one is planned in Belledonne. The analysis of land cover dynamics over the 1998–2009 period showed that the region had undergone steady urban growth with extension of residential and industrial areas at the expense of agricultural land, mostly in the Grésivaudan valley and the north-western Bièvre and Voironnais areas [24].

### 2.2 Recreation service models for the mountains vs. lowlands

Mountains are strongly attractive to urban and peri-urban users. They provide a sense of escape, a feeling of wideness and wildness which draws people from afar regardless of travelling cost. The mountain ranges of the Grenoble region are no exception to this rule. Standing at the entrance of the Alps, they are also the most accessible mountain ranges for out-of-region population. During week-ends visitors come from as far as the Lyon urban area, more than a hundred kilometers away, for day or overnight trips.

Mountains and valleys appeal differently to the different local populations. In a study of leisure mobility, Rech et al. [21,25] identified two types of recreationists among the population of the Grenoble urban area. Sixty nine percent of respondents were qualified as hyper-mobile: they travelled more often, longer distances for both work and leisure, tended to spend their leisure time outside the urban area, and practiced outdoor sports; they were aware of the presence of mountain protected areas and visited them regularly. By contrast, non-mobile respondents (31%) travelled less, spent their leisure time within the urban area, and did not practice outdoor sports. Mountain areas within the immediate vicinity of Grenoble were the most visited natural areas, Belledonne being more popular in winter, while Chartreuse and Vercors were more visited in summer. In all cases proximity was identified as a key factor for the choice of destination [21,25]. Additionally people may choose nearby locations in the valleys for their after-work leisure, but will be more prone to escape to the mountains during the weekend for outdoor sports.

Due to this dichotomy of the landscape in the study area–on the one hand remote but attractive mountains with outstanding natural landscapes and scarce population, and on the other hand highly populated agricultural lowlands, highly accessible but with a more ordinary nature–a representative model of recreation service ought to comprise two sub-models.

We therefore considered upland vs. lowland separately. We spatially segregated them following an elevation threshold set at 500 meters, which captured a continuous plain area along the Isère valley and the northeastern lowlands. The Chambaran and Voironnais, two hilly but non-alpine areas, were incorporated into the plain and valley model. Conversely the Monteynard-Avignonet Lake, which forms a canyon, was included in the mountain model as its elevation was not representative of its identity.

### 2.3 The Grelou model–an application of the recreation opportunity spectrum

**Fig 2:**
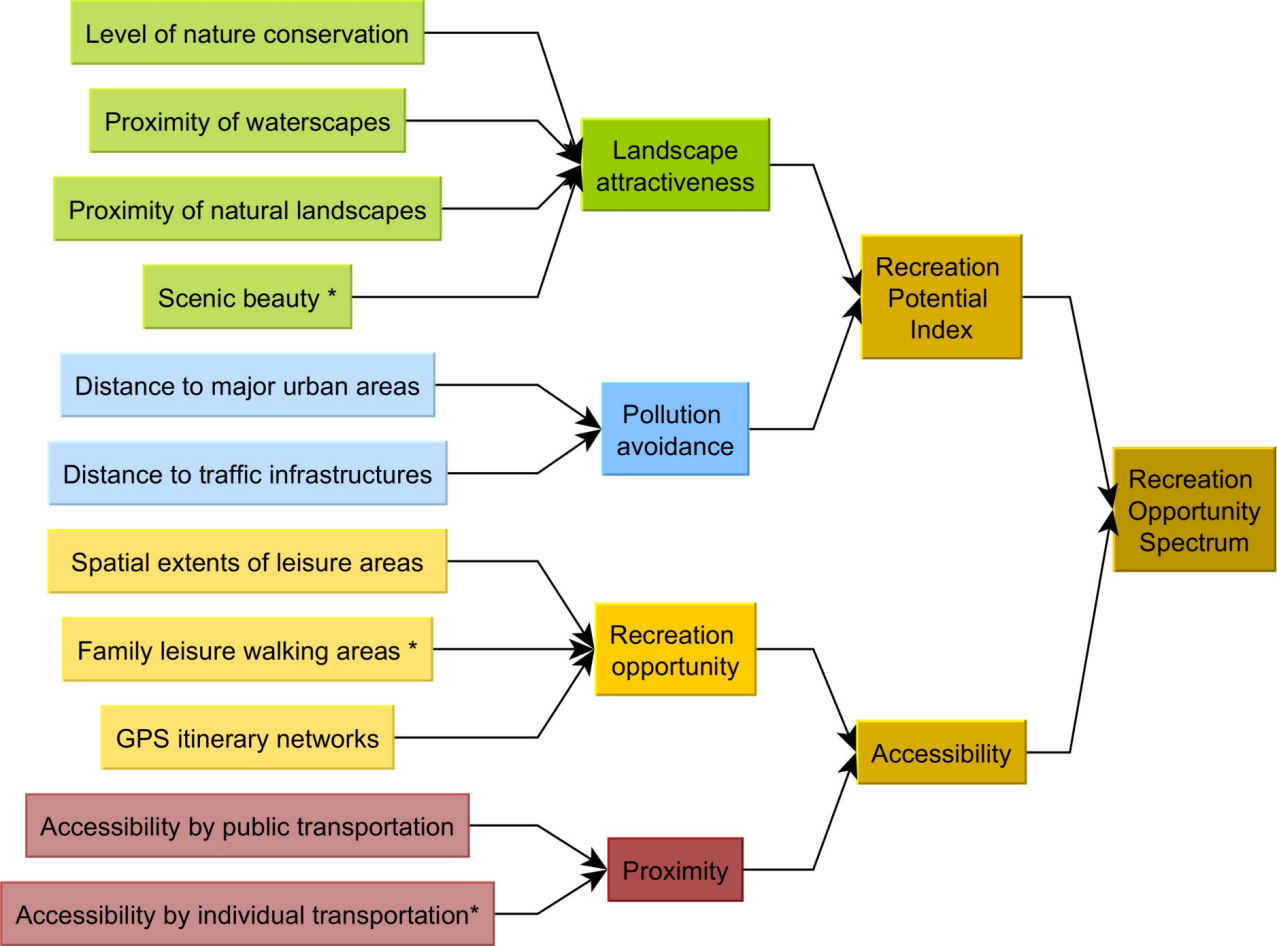
Structural diagram of the recreation opportunity spectrum model applied for the Grenoble urban area–the Grelou model. 12 indicators (left) were used as proxies to assess the 4 main factors (middle) of the Recreation Opportunity Index and the Recreation Opportunity Spectrum (right). Factors marked with an asterisk * were computed differently in the two sub-models (upland vs. lowland).

The Recreation Opportunity Spectrum model [10] can be used to estimate the recreation ES by combining the provision of recreation opportunities by ecosystems with their accessibility [9,14]. We assessed the Recreation Potential Index as the product of a composite landscape attractiveness factor and a second factor accounting for avoidance of pollution. Accessibility was quantified as the product of a remoteness vs. proximity factor and of a factor of availability of recreation infrastructures (Fig 2). We selected the spatial indicators for our case study following a literature review [5,9,13,26] of preferences for recreation, and according to their relevance in the Grenoble context.

We estimated landscape attractiveness as the sum of (1) the proximity of natural landscapes, (2) the presence of protected areas and the level of nature conservation in general, and (3) the proximity of waterscapes. In the mountain model, we added a landscape scenic beauty factor (following [27]) in order to account for the attractiveness of panoramas offered by mountain landscapes in open areas.

We calculated the total avoided disturbance as the minimum of two factors of distance to sources of pollution: distance to urban areas which induce air and visual pollution, and distance to roads, railways and airfields which cause noise pollution. These disturbances were prominent in the valley and plain areas, but rather limited in the mountains. However these factors could play differently in a scenario analysis, as urban sprawl might affect semi-urban mountain areas (i.e. inner valleys of Chartreuse and Vercors) in the coming decades.

We mapped recreation opportunities for site-based and itinerary-based outdoor activities. Site-based activities were spatially discrete and easily mapped: e.g. climbing sites, river banks, lake-based leisure areas and multi-activity resorts. Itinerary-based activities were spatially diffuse and could not be mapped so simply. We modelled their fine-scale spatial extents using GPS tracks downloaded from sport-oriented crowd-sourced websites. Although contributors to GPS track repositories are mainly medium- to high-level practitioners, these websites are extensively used by regular recreationists, and greatly influence their choices of destination. We thus considered that they represented a reliable proxy for visitation opportunity and could thus reliably be used for recreation modelling. For this study, we used crowd-sourced sports-oriented websites to build opportunity networks for ten sports: hiking, trail running, mountain biking, cycling, skiing, snowshoe-hiking, multi-pitch climbing, mountaineering, ice-climbing and horse-riding. We also included a series of non-GPS-track-based models in order to account for other major leisure and recreational activities: climbing areas, family leisure-walking along river banks and lake shores, as well as leisure activities and nautical sports around lakes. Compared with the first series of sports, these activities are much less spatially diffuse and challenging in terms of natural area management, but are nevertheless essential for assessing recreation ecosystem services since they concern a much larger proportion of the population [5,13].

We estimated proximity vs. remoteness as the number of people who were likely to use a recreation site or itinerary, weighted by the distance to be travelled to reach the relevant access points—parking areas and bus stops. To better reflect differences in attractiveness, we restricted the accessibility of sites with decreasing maximum distances for (1) mountain areas, (2) a selection of major recreation sites in the lowlands, and (3) more ordinary plain and valley areas.

Consequently, the recreation ES was estimated using the following equation:

$$\begin{array}{l} \;ROS=Recreation\;Potential\;Index\times Accessibility\\ \;=(\sum landscape\;attractiveness\;factors)\times (\min\left(pollution\;avoidance\;factors\right))\\ \;*\left(\sum recreation\;opportunities\right)\times \left(\sum proximity\;vs.remoteness\;factors\right) \end{array}$$ (1)

Some details one the implementation of this modelling framework through Eq (1) are provided in S1 Appendix.

### 2.4 Factor calculations

#### 2.4.1 Recreation opportunities

Site-based activities were modelled based on the location of recreation sites, whereas all the other considered activities such as hiking were itinerary-based and modelled using GPS tracks. Only generic features of the modeling are described here. Quantitative details can be found in the Supplementary Information as indicated along the text.

Three major recreation activities were not or could not be included: caving, paragliding and canyoning (see S2 Appendix).

**2.4.1.1 Recreation opportunity maps for itinerary-based activities**. For itinerary-based activities, we retrieved GPS tracks from a selection of crowd-sourced websites: skitour.fr, vttour.fr, visugpx.com and camptocamp.org (see S1 Table). These websites are commonly used in the region. They offer a high-quality and dynamic service in terms of number of referenced sites and availability of georeferenced information. They were therefore considered a reliable input for mapping outdoor activity itineraries. See S3 Appendix for quantitative details.

**2.4.1.2 Recreation opportunity maps for site-based activities**. The activities modelled here encompass climbing, lake-based leisure and leisure activities on river banks such as walking, cycling and jogging.

We downloaded GPS coordinates of main climbing sites from the crowd-sourced website camptocamp.org, considered as a reliable data source as it is a national reference for mountain sports and climbing in particular. We mapped lake-based leisure activities based on the national database of water bodies (BD CARTHAGE^®^; http://professionnels.ign.fr/bdcarthage) and using as a source of information a tourist guidebook distributed by the regional tourism administration (Isère tourisme, 2014; www.residence-silenes-allevard.com/pdf/documentation-lacs-fr.pdf). Fifteen lakes and water-bodies in the study area were listed with a list of possible recreational activities provided for each of them. The banks of the Isère and Drac rivers are extensively used by the local populations for leisure. We assumed that all sports activities would be already accounted for in the GPS opportunity networks, and thus only considered leisure, walking and jogging. We filtered streams suitable for leisure using a slope threshold set at 10°. See S4 Appendix for more quantitative details.

#### 2.4.2 Attractiveness-weighted remoteness

We computed accessibility of recreation sites and itineraries as the total population of the municipalities from which visitors are likely to originate, divided by the distance to be travelled from each of these town centers to reach the access points to the considered sites. This choice for the distance dependence is qualitatively expected, albeit quantitatively somewhat arbitrary.

For individual transportation, we considered as access points: parking areas near mountain recreation sites and trail networks (GPS coordinates downloaded from camptocamp.org), parking areas of lowland recreation sites, and lowland town centers when no particular recreation site was found. See S5 Appendix for more details.

#### 2.4.3 Landscape attractiveness

**2.4.3.1 Proximity of natural landscapes and waterscapes**. We hypothesized that landscape attractiveness was first correlated to the proximity of natural land covers or waterscapes [13]. We included in the first category all grassland and forest types, as well as shrubland and bare areas (rocks and glaciers) in mountains. As the proximity of water plays a particular role for enjoying nature, we considered it separately. Consequently, proximity to both factors made the landscape twice as attractive as the proximity to a single one. See S6 Appendix for more details.

**2.4.3.2 Level of nature conservation**. We hypothesized that besides the presence of natural land covers, wellbeing during recreation time was correlated to the level of nature conservation [13]. We assumed that the gradient of environmental protection between urban areas on the one hand and protected areas on the other can be assimilated to a gradient of potential human impact and of overall naturalness (of opposite sign). We estimated this gradient using various official zonings and inventories. See S7 Appendix.

**2.4.3.3. Scenic beauty (mountain only)**. We assessed the perceived scenic beauty of mountain landscapes using a viewshed analysis combined with measures of landscape heterogeneity following the model developed by Schirpke and coworkers [27]. Although perceptions may vary among individuals [28], based on survey data, this model relates scenic beauty, or aesthetic value, to indicators of landscape structure. The details of the viewshed model are presented in S8 Appendix.

#### 2.4.4 Avoidance of disturbance

**2.4.4.1 Avoidance of noise pollution**. The avoided noise pollution factor was assessed for road and rail traffic. In all cases noise pollution avoidance was considered to be maximum beyond the maximum impact distance, zero within half this distance, and to vary linearly between these two thresholds. We used maximum impact distances of noise pollution for different categories of roads and railways (tram and train lines) obtained from the Rhône-Alpes region online data center (http://www.georhonealpes.fr). These values ranged from 10m for a small road to 300m for a highway or a high speed train railway. Likewise we set a noise pollution distance of 500m around the Le Versoud airfield takeoff and landing sites. We considered noise pollution due to the local airport in Saint-Geoire to be negligible since it functions only on winter weekends.

**2.4.4.2 Avoidance of air and visual pollution**. We considered the immediate vicinity of major urban areas to reduce the Recreation Opportunity Index due to visual and air pollution. For this factor we retained urban patches exceeding 400ha, a threshold which allowed isolating all major urban clusters. We computed avoidance of urban areas to be zero within these areas and to increase linearly until a maximum distance of 500m beyond which it remained equal to 1. The distance is an estimate for building height to have negligible impact on the view.

## 3. Model analysis and validation

### 3.1 Analysis of model outputs

To identify clusters of high and low ES values–hereafter referred to as hotspots and coldspots, we ran a cluster analysis the Getis-Ord Gi* algorithm [29,30] in ArcGIS Spatial Analyst. For this purpose the output map was re-aggregated at a resolution of 250m x 250m. This analysis was run for the two sub-models together and independently,–with confidence levels of 99%, 95% and 90%.

We then validated our model by confrontation with the results of the InVEST recreation ecosystem service model against the results of an online survey.

### 3.2 Comparison with the InVEST recreation model

The InVEST recreation model [17,18] performs a regression analysis over any study area using as a response variable the number of georeferenced pictures posted on the social media website FlickR, and any shape indicator as explanatory variables. The model allows users to perform the regression analysis using their own GIS data as explanatory variables, or to use default data provided by the software. However the model is restricted to linear regression, only accepts shape data as input, and cannot process quantitative data unless forced by using a time-consuming procedure. Consequently and after confirmation by the developers we retrieved the response variable data–i.e. the map of picture density–and performed the analysis outside of InVEST, using R core software [31].

Due to the presence of very strong outliers (pixels with an abnormal density of georeferenced pictures) in the FlickR dataset we removed their upper centiles from the analysis. These outliers were mostly concentrated in the Grenoble city center and in ski resorts, with a large part of selfies among these. Some other outliers matched high-profile events like music festivals or *Coupe Icare*, one of the world’s biggest free flight meetings. Finally some outliers were found in unexpected places and due to FlickR ‘super-users’ who would post hundreds of pictures of their daily lives on the website.

The comparison of Grelou with InVEST estimates requires some explanation. Due to their different natures, the direct comparison of the model outputs is meaningless. Grelou is a Spatial Multi-Criteria Assessment based on *a priori* variables designed to evaluate recreational potential, while InVEST is a regression model evaluating the relevance of hypothetical explanatory variables for observed visitation data. In practice, we used the Grelou *a priori* variables as explanatory variables for InVEST. We then analyzed both the relevance of these variables within the InVEST conceptual framework, and the relevance of InVEST estimates in the context of the Grenoble area.

We tested several linear models and generalized linear models for mountains and lowlands separately using the factors we developed for Grelou as explanatory variables for InVEST outputs. All explanatory variables were standardized by mean and standard deviation. We selected the best model using the AIC criterion [32]: a Generalized Linear Regression Model with a Poisson distribution–which was appropriate for the modelling of ‘rare’ events such as the map of georeferenced pictures. We then performed variable selection using a stepwise regression analysis and two additional Anovas (type II).

### 3.3 Online survey of recreational practices

We performed an online survey of recreational practices to validate our model. The detailed questions can be found in S9 Appendix. The survey was designed to assess several questions organized as four steps:

1. the respondents’ social profiles and recreational habits
2. the contribution of the environment to their experience during recreation time
3. the factors which influenced their choices of destinations for recreational activities
4. the factors which would induce a change in their recreational habits in the future

Following Kienast and coworkers [26], our survey included an interactive map of main geographic units within the study area (based on [24]). The respondents were asked to select among 54 such units those they had visited for recreational purpose. The map came along with an interactive description of main districts and a list of high-profile destinations within each of them to orient respondents when they wouldn’t recognize districts from the map and name only.

The rankings of different factors within steps 2–4 were tested using the Friedman and Nemenyi tests.

## 4. Results

### 4.1 Influence of model factors on the spatial structure of Grelou outputs

**Fig 3:**
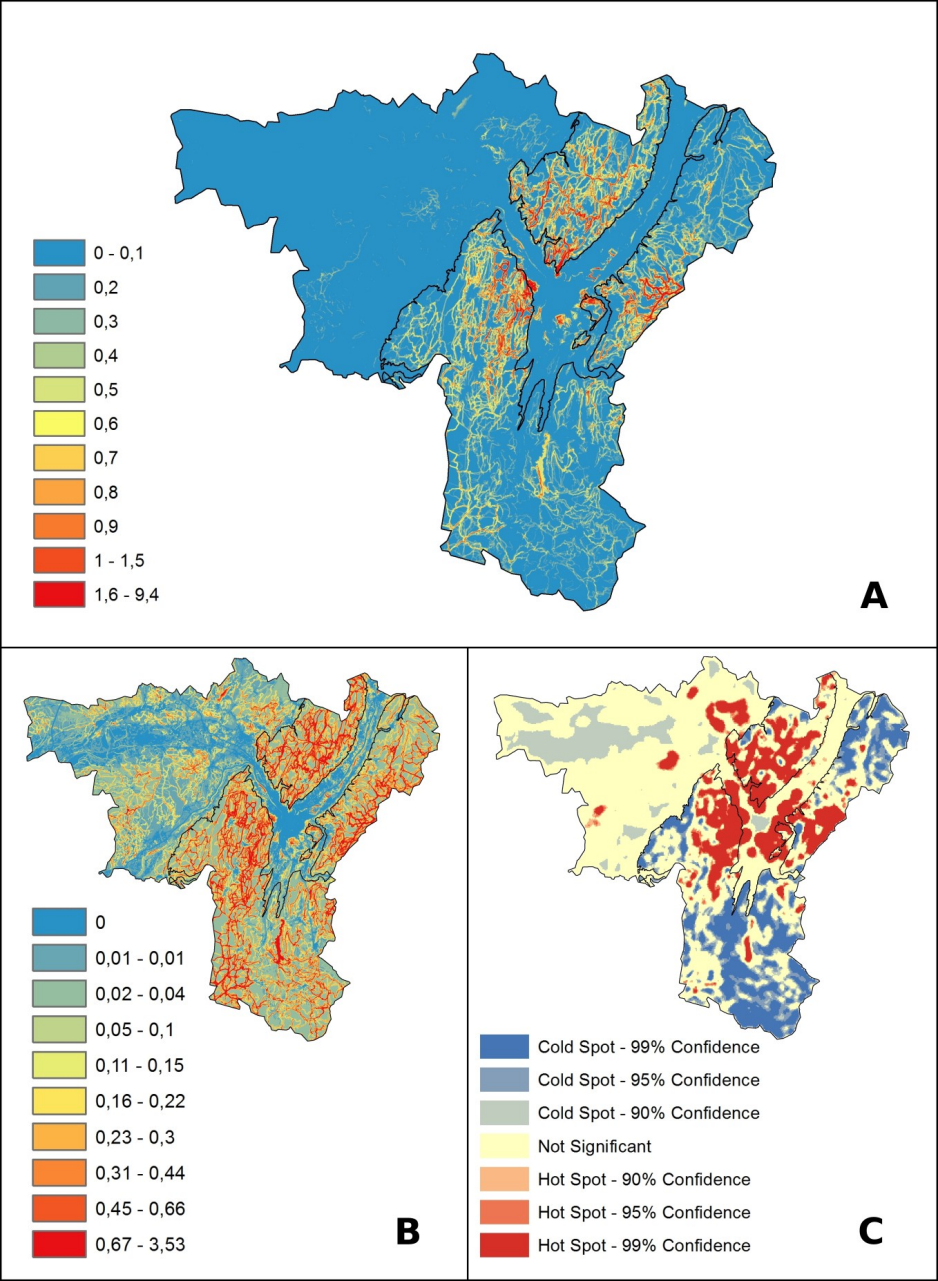
Grelou output maps. ES values are discretized by equal intervals (A) stressing the predominance of mountain areas, or deciles (B) enhancing spatial variations. C: map of hot and cold spots obtained from the Getis-Ord * cluster analysis.

The Grelou model quantified the recreation ES as the combination of Potential Recreation supply provided by the ecosystems, accessibility of recreation sites and the level of recreation opportunity in terms of number of practicable sports and leisure activities. Estimated ES values ranged from 0 to 10, but only 0.17% of the pixels had a value above 2; these high-value pixels were concentrated on three high-profile hikes (Fig 3A). Mountains had higher ES values than lowlands. Different factors structured the model output at different spatial scales.

The Opportunity Network factor caused ES values to vary strongly at a scale of a few tens of meters. This was an effect of the distance limit around recreation sites and trails which we set at 90 m–an estimated threshold accounting for constraints of distance in mountain areas, given the topography. At landscape scale, the Opportunity Network identified areas of multi-activity opportunity along river banks, in lake-based recreation areas, and in some trails in the mountains which represent some of the most famous hikes.

The landscape factors caused ES values to vary at both the scale of the study area and at landscape scale (S1 Fig). Mountains always displayed a higher recreation potential than lowlands due to the overwhelming dominance of natural areas in the former and of urban and agricultural areas in the latter. At landscape scale the scenic beauty factor caused open areas to stand out in mountains (S1A Fig). In lowlands, urban areas were all estimated to have a ROS value of 0 due to the model focus on the role of ecosystems on recreational activities; and conversely to the ‘urban pollution avoidance factor’ which was zero in urban areas and increased with distance (S1C Fig). On the other hand patches of natural areas close to Grenoble but outside the impact distances of urban areas were all identified as hotspots for the recreation ES.

Lastly, the population-distance factor influenced the result at the scale of the whole study area (S1D Fig). This was due to the dominance of the Grenoble city, which completely polarized the map. The pressure from the populations of nearby major cities outside the perimeter of the study area (Chambéry, Valence, Lyon) was less noticeable. Consistent with results from [21], the areas of northern Vercors, southern Chartreuse and southern Belledonne, which were closest to Grenoble, appeared as the most prominent recreation service-providing areas. Conversely more remote areas with similar levels of opportunity networks and recreation potential had lower estimated ES values. This was the case for Trièves, Matheysine and northern Belledonne.

### 4.2 Spatial extent of recreational activities

The pooling of GPS tracks by sport produced a total of ten GPS networks (S2 Fig). As expected, the spatial extents of these sport-specific networks showed characteristic signatures of these practices. Mountain-related sports such as skiing and snowshoeing were located in mountain ranges only. Climbing-related sports were very spatially discrete, and in steep and elevated areas. Cycling was found mainly in the road network. Some activities such as trail running and mountain biking were present in upland and lowland alike.

In principle, the use of opportunity networks such as these enables the assessment of landscape multifunctionality in terms of diversity of opportunities for recreation activities. However the sports we considered did not all play similar roles. The five activities associated to the road and trail networks: hiking, mountain biking, trail running, cycling and skiing, had overlapping opportunity networks (S2 Fig). These did not spatially overlap with climbing-type activities, which were associated to different kinds of landscapes and also overlapped little among each other.

Some activity-specific networks were more close-knit than others (S2 Fig). To some extent this reflected differences in popularity of these activities but this was biased towards performance-oriented practices whose adepts are more prone to using GPS tracks. This was especially true for the mountain biking network which represented a third of the total number of GPS tracks (S1 Table). Additional work would be needed to quantify this bias to estimate visitation rates. Nevertheless, in a case where the analysis is limited to a single activity, the number of GPS tracks can provide an estimate of visitation rates or at least of their spatial variations over the area. We calculated the total length of recorded GPS track per cell of 250m × 250m separately for each sport (examples shown for hiking, skiing, mountain biking and trail running, see S3 Fig).

### 4.3 Cluster analysis

At the scale of the whole study area, hotspots were concentrated and larger in mountains whereas lowlands hosted mostly large areas identified as coldspots, along with a few small hotspots (Fig 3C). The cluster analysis within each of the two zones aimed to remove their qualitative differences. It identified the same hotspots within mountains but with a slightly smaller spatial extent, while the plain model identified more numerous and more extended hotspots, and the spatial extent of coldspots decreased considerably. The major hotspots were located in mountains immediately surrounding the Grenoble urban area, covering nearly all of southern Chartreuse, southern Belledonne and northern Vercors. In particular, each of these areas hosted one of the three major hikes which concentrated the highest ES values. Additional hotspots were found in smaller areas, including lake-based leisure areas, and around Mont Aiguille, a very popular destination for all types of climbers.

In lowlands, all natural areas in the vicinity of Grenoble were identified as hotspots. They included portions of the Isère river banks, a lake-based recreation area, forests and hilly plateaus with traditional agricultural landscapes. After restricting the analysis to lowlands, several additional areas were identified as hotspots, corresponding to lake-based leisure areas, natural landscapes near the medium-sized town of Voiron, and the forest and plateau of Chambaran, in the hilly north-west areas. These hotspots were therefore not major service-providing locations at the scale of the whole study area, but were used for more ordinary and local after-work-like recreation. In addition, small coldspots were identified in the core of the Grenoble urban area and in the agricultural plains of Bièvre-Valloire.

### 4.4 Comparison of the Grelou vs. InVEST models

#### 4.4.1 Consistent effects of landscape attractiveness and recreation opportunity

The Grelou recreation opportunity networks had significant and positive effects for explaining the spatial patterns of FlickR georeferenced pictures density (i.e. the proxy variable for observed visitation) through InVEST, in both the mountain and lowland cases (Table 1). Hiking, cycling, skiing and trail running networks had the largest effects in mountains. Hiking, cycling, mountain biking and family strolling contributed most in lowlands. In mountains the presence of protected areas and scenic beauty had large effects, which was consistent with the presence of very scenic panoramas in mountain open areas, namely in the Chartreuse and Vercors regional parks.

**Table 1:** Results of the general linear model of visitation estimated by InVEST using Grelou factors as explanatory variables. Only factors with a significant effect are presented. Spatial indicators are grouped according to their ROS factor category: landscape attractiveness (in green), disturbance avoidance (in blue), recreation opportunity (in orange) and proximity of sites to populated areas (in red). Significance levels account for p-values and related codes: 0 ‘***’ 0.001 ‘**’ 0.01 ‘*’ 0.05 ‘.’ 0.1 ‘ ‘ 1.

	Mountains			Plains and valleys		
	Estimate	Std. Error	sign.	Estimate	Std. Error	sign.
(Intercept)	-2.259	0.018	***	-2.713	0.023	***
Natural landscapes	-0.105	0.015	***	-0.159	0.017	***
Scenic beauty	0.189	0.014	***			
Protected areas	0.133	0.021	***			
Water proximity				0.107	0.021	***
Urban avoidance	-0.132	0.013	***	-0.499	0.021	***
Noise avoidance	-0.047	0.010	***			
Hiking	0.304	0.016	***	0.134	0.012	***
Cycling	0.211	0.013	***	0.206	0.014	***
Skiing	0.257	0.012	***			
Trail running	0.138	0.015	***	0.066	0.012	***
Mountain biking	0.070	0.016	***	0.194	0.018	***
Water stroll	0.028	0.012	*	-0.103	0.02	***
Multi-pitch climbing	0.031	0.008	***			
Climbing sites	0.057	0.009	***			
Snowshoe-hiking	0.023	0.007	**			
Lake leisure	0.078	0.010	***	0.079	0.009	***
Proximity (bus)	0.088	0.015	***	0.057	0.017	***
Proximity (car)	0.087	0.017	***	0.140	0.019	***

#### 4.4.2 Opposite effects of urban-related factors as source of mismatches between models

Avoidance of urban-related disturbances had strong negative effects for explaining the spatial patterns of FlickR georeferenced pictures density in both mountains and lowlands. This was also the case for the proximity of natural areas, although to a lesser extent. This reflected the opposite behaviors of InVEST and Grelou for urban areas–and consequently of natural areas with which they are spatially mutually exclusive. Indeed Grelou did not consider urban areas as outdoor ecosystems, and thus imposed a null ecosystem service in these areas. On the contrary, InVEST measured recreation through the local density of FlickR pictures, which in our area was highest in urban areas. A review of hotspots of FlickR pictures revealed that they were mostly pictures of city life, of high-profile events such as music festivals and sports contests, or panoramic pictures of the surrounding mountains taken from town centers, and were then mistakenly associated with the recreation ES.

Second, proximity vs. remoteness of recreation areas did not have large effects in the Grelou model for mountains. Grelou assumes that distance between settlements and local outdoors is critical for explaining visitation potential. However this effect was not found in InVEST, due to the presence of picture hotspots related to high profile events taking place in remote areas. Although these events were not related to the recreation ecosystem service, they caused an overrepresentation of the spatial patterns of FlickR georeferenced pictures density.

#### 4.4.3 Map comparison and confusion matrix

**Fig 4:**
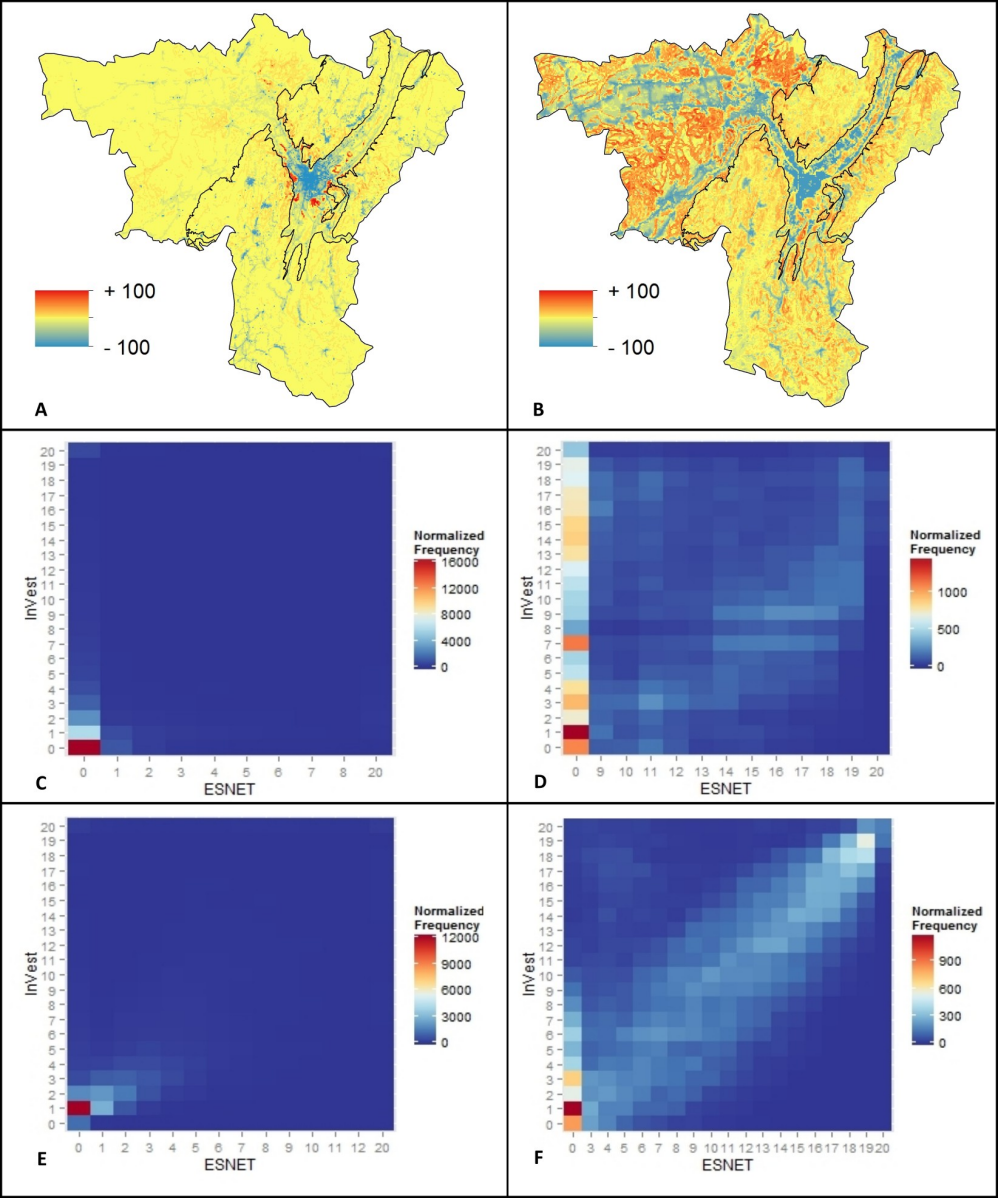
Compared predictions of Grelou (x axis) and InVEST (y axis) models. Differences between model outputs (A,B) standardized by their respective maximum values. Blue tones indicate higher predicted values by InVEST; red tones indicate higher predicted values by Grelou. Confusion matrices for the lowlands (C,D) and the mountains (E,F) independently. Results are presented in absolute values (A,C,E) and in deciles (B,D,F).

Due to the opposite roles of urban areas in Grelou and InVEST, the predictions for recreation ES in lowlands were opposite. The comparison maps and confusion matrices show that each model predicted high ES values where the other would predict low ones whether in areas of high or low ES values (Fig 4). In areas of high ES values, the two models thus identified mutually exclusive hotspots.

In mountains where urban areas are more spatially restricted, there was a better level of agreement between the two models, although InVEST still predicted high ES values in town centers and along main roads, where Grelou would impose low values. The Grelou model predicted higher values than InVEST in areas corresponding to ordinary nature.

### 4.5 Online survey validation

The profile of the respondents to our online survey is discussed in S10 Appendix, as well as the use we have made of this online survey to validate our outputs. Here, we focus on the perception of ecosystem services as captured by this survey.

When asked about factors motivating outdoor recreation respondents discriminated significantly across all proposed factors (S4A Fig), with the following ranking: 1: ‘contact with nature’, 2: ‘sport practice’, 3: ‘air change’, 4: ‘calm’, 5: ‘touring’.

Second, consistent with previous results, they identified as most important benefits of their outdoor recreation those linked with health and aesthetic experience (89% and 76% of votes respectively, (S4C Fig). Social benefits ranked third, but this may be underestimated since 40 people expressed in the free expression section the importance of shared experience with relatives–which they had not identified with the definition of ‘social benefit’. Other proposed benefits, more linked with cultural amenities, were identified as less important (educational: 22%; cultural: 15%, artistic: 8%; spiritual and recreational: 0%). These results were consistent with the findings by [21] in the same region.

These findings indicate a strong perception of the recreation ecosystem service, in the sense that the link between wellbeing and nature was clearly expressed. This connection was mostly associated with health benefits and sport practice, which was an expected result given the population targeted by the model and the questionnaire.

#### 4.5.1 Validation of model hypotheses

We then confronted respondents’ answers to model hypotheses regarding: (1) the relevance of separate parameterizations of lowlands and highlands, (2) the relevance of the selected structuring factors, and (3) the even weighting of each factor. To discuss these points, three questions bore on (1) the respondents’ leisure habits, (2) the important factors in their choice of destination, and (3) their landscape preferences.

To the question of leisure habits, 75% respondents had different practices during weekdays and weekends. 50% declared a change in leisure location–staying near home during the week and going farther in the weekend; 30% a change in practiced activities, and 23% declared to practice alone during the week, and collectively during the weekend. These results, in association with the spatial extents of sport-specific networks, support our hypothesis of different uses of lowland and mountain areas, for the after-work daily leisure and weekend trips respectively.

We asked respondents to rank a list of factors according to their importance in selecting leisure destinations. We found three statistically different groups of factors (S4B Fig). Panorama and nature conservation were ranked as the most important factors. Landscape features/oddities, distance to urban disturbance and types of landscapes came second, and travelling distance last.

When asking to detail their preferences for certain types of landscapes by selecting among a list which they sought for their leisure (S4C Fig), respondents preference scores followed a gradient from heavily human-impacted landscapes (selected by less than 15% of respondents), to natural and semi-natural landscapes (24 to 29% of votes), and finally to waterscapes and mountain-specific landscapes (49 to 85% of votes).

Respondents indicated that panorama was the most important factor for selecting a destination, which validated our inclusion of ‘scenic beauty’ as a primary factor of landscape attractiveness. The second most important factor was ‘a nature in good state of conservation’, which we accounted for through two factors: ‘conservation status’ and ‘proximity of natural landscapes’.

Then, respondents ranked ‘the presence of a particular landscape feature/curiosity’, ‘the presence of particular landscape’ and ‘distance to urban areas’ as second-order factors in the choice of destination. However, when asked to detail their landscape preferences, they expressed a marked preference for mountain landscapes and waterscapes, and to a lesser extent for other natural and semi-natural landscapes, and a strong dislike for urban, peri-urban landscapes and open field views. This was consistent with our hypotheses that mountains were more attractive to visitors and that waterscapes played a particular role in landscape attractiveness.

The presence of a particular landscape feature (geological or landscape curiosity) was also ranked as a second-order factor and should therefore be included in the model. Indeed many hikes are named after a visual curiosity present on the trail and certainly contribute to the choice of destination. However to our knowledge no such list exists and building this database from pre-existing information was not feasible within our study.

Travelling distance was on average perceived as the least important factor in choosing a destination. Several elements may help interpret this result. First the notion of proximity was not detailed in the questionnaire (in terms of kilometric distance). In a study of mobility of Grenoble populations for leisure [21], proximity was a key element for the choice of destination, but at scales largely exceeding the size of the study area: mountain ranges surrounding Grenoble were highly preferred to more remote ones. Second due to the region’s topography, travelling times are mostly influenced by reaching the mountains rather than going far: all mountain destinations within the study area are within 30 to 60 minutes driving from Grenoble, probably influencing the perception of distances. Overall, at large scale, proximity is a key factor with recreation trips being intrinsically limited to a 100-200km distance, but within our study area practitioners may be less sensitive to travelling distances making landscape attractiveness more important as a selection factor.

## 5. Discussion

Concerns have been recurrently expressed in the literature about the best modelling strategy for cultural ecosystem services, which is required for analyzing their spatial relationships with other ecosystems services and for their integration into environmental assessments, land management and planning [2,3,33]. Using GPS tracks downloaded from crowd-sourced websites can address these concerns [5,34]. Here, we have developed a novel standardized methodology to assess simultaneously recreation ES associated with multiple outdoor activities. This approach may also be used to better study the environmental impacts of outdoor sports [35] and to foster a more sustainable management of recreation areas [36].

### 4.1. Addressing the challenges of modelling recreation ES

Cultural services are a long-standing challenge for ecosystem service researchers [4]. Although they figure prominently in ES typologies, they have proved difficult to model, quantify and spatialize. Cultural services are associated with personal experiences of interaction with nature, and are thus related to a diversity of intangible values and immaterial benefits [28,33,37]. Perceived benefits also depend on individual preferences, which often relate, at least to a large extent, to cultural and social factors [28,38]. Of all cultural services, the recreation ES may be easiest to quantify through visitation, and is indeed the most commonly quantified cultural ES [6]. Recent studies have also tried to disentangle its different dimensions, from supply capacity, to demand and flow [8,39].

However, the recreation ES encompasses a whole range of practices, perceptions and benefits. The Recreation Opportunity Spectrum (ROS) [10] concept, originally developed in the 1970s for the management of recreational activities in US national parks [40], aims to capture such complexity. ROS studies have described the multiplicity of visitor uses and expectations including the degree of desired wilderness, loneliness and personal challenge, or the demand for facilities, ease of access, and family or social-bonding experience. These environmental, social and managerial expectations vary between [19,41,42] and among [43] visitor categories and constitute as many considerations for managers and land planners to satisfy a diversity of visitor profiles. A special attention has been devoted to this diversity of visitor profiles in the recent decades in the French Alps, where recreation and tourism outdoor activities have boomed, both regarding overall activity and in the development of new practices [22,44]. These recent developments have stimulated new environmental, economic, social and political concerns for sustainability, including the management of environmental impacts and of interactions between various stakeholders [22,25].

Capturing the diversity of recreational outdoor activities raises the question of the choice of relevant spatial indicators; [6] found that only 23% of all articles on cultural ES modelling provided a map. Spatial indicators included visitation rates (number of visitors or visitor-days), site/facility/attraction mapping, or recreation/tourism-associated expenditure, or travel cost indicators.

In response to these challenges, Grelou’s original approach specifically targets the recreation ES through the precise and specific tracking of the presence of recreational visitors. By using GPS tracks downloaded from crowd-sourced websites, we developed a standardized methodology which addresses in a single run a large range of outdoor activities. Although the use of GPS track recording might be more characteristic of challenge-seeking recreationists, the resulting visitation network constitutes a maximum spatial extent of visitation, and thus also *includes* the locations visited for less intense practice. As such, Grelou emphasizes recreational multifunctionality of outdoor areas, a critical perspective that has not been considered in previous studies. For simplicity we used a common set of landscape preferences for all users, although the literature supports heterogeneous motivations [41–43]. However, some sport-specific preferences are *de facto* expressed through the spatial signatures of the visitation networks [45]. A further analysis of the online survey may determine whether specific preferences regarding the landscape factor or willingness to travel exist depending on sports, social groups or levels of practice.

Regardless of the representativeness of our model of recreationists based on their level of practice or recreation preferences, GPS track networks target practitioners of ‘modern’ and ‘postmodern’ outdoor activities, *i*.*e*. all traditional mountain sports and their newest forms and hybrids [22]. Our model is therefore most probably biased towards the wealthier social classes–a phenomenon identified as a ‘vertical segregation of social classes in mountain practices’ [46]. This raises the question of social inequities in the management of recreation ES [19].

In order to compensate for this bias, we included activities that are typically not associated with GPS technology such as family walking, jogging or lake-based activities. Although we modelled these activities in less detail than the GPS-track-based ones, including them by broad categories was enough for hotspots of non-sportive recreation to stand out in the final output.

Also, our model is targeted at recreation purposes only. It should be complemented by other perspectives, such as non-use cultural values, which would be relevant for a larger public of beneficiaries including people who do not practice outdoor activities, and sports practitioners who may also value areas that they visit–or not–beside their recreation potential.

### 4.2. GPS track networks are an accurate spatial indicator of personal experience

This study demonstrates how GPS track networks provide useful insights for recreation mapping and combine advantages of other indicators.

First, although in the current state of development of Grelou, GPS track networks are not calibrated to directly assess visitation rates we found that the predicted multifunctional areas strongly correlated with the visitation rates estimated from declarations of respondents to our online survey. This gap could be addressed by considering the number or length of GPS tracks (and not only the number of activities). However we would expect that such an indicator would be biased between sports (challenging activities being more prone to the use of GPS tracking), as well as within each activity (e.g., towards emblematic races).

Second, site, facility and attraction mapping provide accurate information on recreation potential but with no guarantee of actual use (or ES flow [8]), and depend on the availability of relevant, representative and thus labor-intensive GIS data. More importantly, maps of available trails for hiking, biking or skiing only inform on their theoretical and official purpose. The universal format of GPS tracks allowed us to easily include them in GIS models, to pool data from multiple sources and to model all activities using a single standardized methodology. This is a valuable asset considering the current blooming of new and hybrid sports.

Third, expenditure indicators commonly used for economic valuation may not exist for all recreation activities and put the emphasis on consumption of associated goods and services more than personal experience. GPS track networks are built from data which imply some high profile consumption habits (GPS device, internet network, specific high-tech sportswear, etc.) but are representative of visitation by a larger public. They allowed us to draw a broader picture of the recreation ES, possibly detailed by activity or identified by groups sharing homogenous recreational profiles: landscape preferences, perceived benefits, or even economic valuation, for instance using data on travel and equipment cost.

Lastly, spatial regression models based on the density of georeferenced pictures on crowd-sourced websites have gained currency as experience-based indicators for the recreation ES [15–17]. Our comparative analysis however suggests that GPS track networks provide better insights into the actual type of recreation than the mere appreciation of the landscape aesthetic value, and provide much more complete and accurate spatial information.

### 4.3. Management implications

Capturing the spatial patterns of cultural ecosystem services enables a better analysis of their spatial covariance or exclusion with other services for multi-ES management and planning. Understanding the environmental or social factors and processes which drive such synergies or tradeoffs would help managers to better target multi-purpose conservation efforts or to enhance the efficiency of their management strategies. The spatial congruence of recreation values with other ES, or with biodiversity in particular, may be an opportunity for multi-targeted conservation, or may conversely call for specific strategies. Spatial congruence may be an opportunity for aligning biodiversity-targeted conservation planning [33, 47] or land plans [48] with the provision of recreational services. Conversely, [49] found little or no spatial correlation in the UK between recreation and other ES or biodiversity, and [50] concluded in the Stockholm region that a dedicated recreation policy may be required additional to conservation policy.

Spatial congruence may also be a source of difficulty if an intrinsic tradeoff [51] hinders multiple goals, e.g., the promotion of recreational activities or tourism and the protection of threatened species or vulnerable landscapes [33,52]. Given such objectives GPS track networks and recreation multifunctionality maps provide accurate spatial information that can be used to analyze potential conflicts, and so help determine appropriate management strategies (access restriction, user information, etc.).

The demand for information about cultural ecosystem services exists at several management and spatial scales [2]. GPS track networks can be used at multiple spatial scales from small regions to nations for studying leisure activities of urban populations in periurban regions. They would however be less useful in very rural areas or countries where GPS data sharing may not be current.

Areas providing high levels of recreation ecosystem services are submitted to a higher intensity and diversity of impacts. Sustainable management must be designed in order to limit the erosion of this resource and the impacts on other ecosystem functions and/or services. For this, documenting and analyzing cultural and social values may help design management strategies congruent with social values [53], raise mutual trust and support among stakeholders and with managers [54], empower local involvement and ensure perennial efficiency of implemented actions [55]. GPS track network-based maps can be used as a resource by communities and outdoor sport associations to inform specific audiences or to illustrate spatial considerations in land planning and management, and foster dialogue among stakeholders. Additionally they may contribute to conflict management among different activities, or between recreationists and other stakeholders).

## 6. Conclusion

GPS track networks allowed us to model with minimum effort and great spatial accuracy the spatial extents of a large range of outdoor activities. Each activity may be considered independently to investigate specific issues such as spatial patterns at local or regional scale, potential impacts on the environment and interactions with biodiversity conservation objectives, or spatial overlap with other recreation or non-recreation activities. This standardized methodology may also be used to assess the recreational multifunctionality of landscapes and thus inform natural area management.

Besides its application in the field of recreation ecosystem services, GPS track networks can benefit several other fields of research and land management including the management of trail networks and the study and monitoring of recreation impacts. They may also provide a spatial support tool to foster dialogue among stakeholders in areas of conflicts.

## Supporting information

S1 Appendix ROS implementation details. (PDF)

S2 Appendix Outdoor activities not accounted for in the Grelou model. (PDF)

S3 Appendix Itinerary-based activities quantitative details. (PDF)

S4 Appendix Site-based activities quantitative details. (PDF)

S5 Appendix Attractiveness-weighted remoteness quantitative details. (PDF)

S6 Appendix Proximity of natural landscapes and waterscapes: quantitative details. (PDF)

S7 Appendix Protection statuses used to compute the ‘Conservation factor’. (PDF)

S8 Appendix Calculation of the scenic beauty factor (mountain only). (PDF)

S9 Appendix Overview of the online validation survey. (PDF)

S10 Appendix Description of results from the online survey. (PDF)

S1 Table Number of GPS tracks or recreation sites per activities. (PDF)

S1 Fig Intermediate factor maps. (PDF)

S2 Fig Maps of spatial extent of opportunity factors. (PDF)

S3 Fig Visitation rates. (PDF)

S4 Fig Results from the online survey. (PDF)
