# Supplementary material for: Benefits of crowd-sourced GPS information for modelling the recreation ecosystem service
Source: PLoS One. 2018 Oct 15;13(10):e0202645. doi: 10.1371/journal.pone.0202645 (PMC6188625; doi:10.1371/journal.pone.0202645)
Supplement: S1 Appendix — (PDF) [file pone.0202645.s001.pdf]

S1 Appendix. ROS implementation details

The qualitative role of the various factors of Fig 2 in the present model is quite intuitive. Furthermore, the general functional form makes also direct intuitive sense:

- The multiplication of factors stems from the fact that all the factors need to be present for a site to present some real recreation opportunity. In other words, the multiplication stands for an "AND" condition in terms of logic: if one of these factors is missing or ranks very little for any given location, its potential is quite poor.
- Conversely for each factor, the various sub-contributions of the various recreation activities contribute independently. In other words, the addition stands for an "OR" condition in terms of logic: any activity can make a site attractive, and the attractivity increases with the number of activities the site hosts. This logic is adapted for cases where the number of activities is not the sole or major contribution to this additive logic.
- The minimum is chosen for pollution avoidance under the idea that the most penalizing noise pollution source is most perceived by recreationists.

On the other hand, the quantitative formulation (both the functional forms chosen and to a lesser extent some of the free parameters in these functional forms) are more arbitrary. This is a major reason why in essence this model represents the potential for recreation, and not a direct predictor of the actual level of frequentation of the recreation sites. In order to transform this potential into an actual predictor, one would need to identify the most sensitive parameters and calibrate them against actual frequentation data[1], a step that is not taken in this work. In spite of this limitation, the correlation with visitation data is satisfactory, as discussed in section 4.

[1] The choice of the functional form is probably less critical, as the functions are used only over a limited range of values; the exact shape in such conditions is of secondary importance.
