# Supplementary material for: Benefits of crowd-sourced GPS information for modelling the recreation ecosystem service
Source: PLoS One. 2018 Oct 15;13(10):e0202645. doi: 10.1371/journal.pone.0202645 (PMC6188625; doi:10.1371/journal.pone.0202645)
Supplement: S2 Appendix — (PDF) [file pone.0202645.s002.pdf]

S2 Appendix. Outdoor activities not accounted for in this model.

Three major recreation activities were not or could not be included in our model: fun-caving, para-gliding and canyoning. We could not include canyoning since the crowd-sourced specialized websites did not offer a downloading option of GPS coordinates of departure and arrival points, but would be feasible in principle. Fun-caving and paragliding are also major activities in the area, especially since the Vercors and Chartreuse ranges are world-class destinations for these two practices. GPS tracks for paragliding and GPS coordinates of cave entrances are available. But although these activities are made possible and enjoyable by air and water circulation and thus general ecosystem functioning, the contribution of ecosystems to their value could not be assessed in the same manner as for other activities. We thus excluded these activities from our model.
