# Supplementary material for: Benefits of crowd-sourced GPS information for modelling the recreation ecosystem service
Source: PLoS One. 2018 Oct 15;13(10):e0202645. doi: 10.1371/journal.pone.0202645 (PMC6188625; doi:10.1371/journal.pone.0202645)
Supplement: S3 Appendix — (PDF) [file pone.0202645.s003.pdf]

S3 Appendix. Itinerary-based activities quantitative details.

For all such activities, we pooled the GPS tracks to create visitation networks, one per activity. In general, these networks follow the road and trail networks, but the GPS networks attest of their effective use independently for each activity. In addition, they can be used to map unreferenced itineraries. We considered visitor presence to diffuse around the line of GPS tracks using a distance function:

$$\mathrm{P_x} = \exp\left(-\frac{d^2(x)}{\mathrm{l}^2}\right) \qquad \text{(S4.1)}$$

where:

- P_x is the probability of visitor presence in pixel x,
- d(x) is the Euclidean distance between pixel x and the nearest pixel on the GPS track network,
- l = 90m is the distance threshold beyond which we considered the probability of presence to decay fast with distance.
