# Supplementary material for: Benefits of crowd-sourced GPS information for modelling the recreation ecosystem service
Source: PLoS One. 2018 Oct 15;13(10):e0202645. doi: 10.1371/journal.pone.0202645 (PMC6188625; doi:10.1371/journal.pone.0202645)
Supplement: S4 Appendix — (PDF) [file pone.0202645.s004.pdf]

S4 Appendix. Site-based activities quantitative details.

To model climbing sites, we extracted from a slope raster at a 15m × 15m resolution areas with a slope greater than 35 degrees in order to isolate cliffs. Then, using the GPS coordinates of sites and pictures from climbing guidebooks, the spatial extents of all wall climbing sites were mapped. Bouldering sites could not be mapped from the 15-m resolution slope raster, in that case a 90m-wide area around the GPS coordinates of the climbing spot was used.

In order to match the activity-counting approach of our GPS track networks for lake-based activities, we attributed each lake a score from 0 to 3, based on the number of types of activities which were possible among the following: (1) leisure areas on the shores, including walking trails, picnicking areas and playgrounds for children, (2) water-based leisure activities, like swimming or canoeing, and (3) nautical sports such as sailing and windsurfing.

Finally, in the same manner as for the GPS networks, we defined a contribution function around the rivers (see S4 Appendix).
