# Supplementary material for: Benefits of crowd-sourced GPS information for modelling the recreation ecosystem service
Source: PLoS One. 2018 Oct 15;13(10):e0202645. doi: 10.1371/journal.pone.0202645 (PMC6188625; doi:10.1371/journal.pone.0202645)
Supplement: S5 Appendix — (PDF) [file pone.0202645.s005.pdf]

S5 Appendix. Attractiveness-weighted remoteness quantitative details.

In order to account for different levels of attractiveness, we hypothesized that visitors could originate from anywhere within a maximum distance radius depending on the type of recreation area they visited. We set these distance thresholds at 10km around town centers in lowland municipalities, 25km around major lowland recreation sites, and anywhere in a 20km radius around the study area for mountain parking places.

In the case of public transportation, we calculated the accessibility of one bus stop as the total population which can access to this bus stop through the bus network, weighted by the distance to be traveled and the line servicing level.

Distance was calculated between departure and arrival bus stops. We limited possible journeys to one bus ride to reach a plain area, and one bus connection to reach a mountain site. We did not consider train transportation except for train lines which replace bus lines in the weekend. Several bus terminals are present in Grenoble and its neighbor cities, which are connected by tramway. We extended the source-population of these terminals to all municipalities which were serviced by the tramway network.

In order to account for the servicing level, all bus timetables in the Itinisère and Grésivaudan networks were analyzed, and weighting factors were applied to their raw population-distance accessibilities using the following rule:

- 1 for high-frequency all-day servicing lines (shuttles)
- 0.75 for a medium-level servicing lines (several stops a day on weekend days and holidays)
- 0.5 for low-level servicing lines (in case of non-service on Saturdays or Sundays or during holidays, low number of stops per day, inconvenient hours for a day trip, or replacement by train).
- 0 for lines which are inactive on weekends and holidays.

The accessibility of an access point AP by car or pus can thus be expressed as follows:

$$a_{AP,tot} = \frac{1}{2}(a_{AP,car} + a_{AP,bus}) \quad \text{(S6.1)}$$

with $$a_{AP,car} = \sum_M pop_M \times d_{AP,M} \times w_{AP,MR} \quad \text{(S6.2)}$$

and $$a_{AP,bus} = \sum_{ML} pop_M \times d_{AP,M} \times w_{AP,M,L} \times freq_L \quad \text{(S6.3)}$$

where:

- $a_{AP,tot}$ is the total accessibility of access point AP,
- $a_{AP,car}$ and $a_{AP,bus}$ are its accessibilities by car and bus respectively,
- pop_M is the population of municipality M,
- $d_{AP,M}$ is the distance between the access point AP and the municipality M,
- $w_{AP,MR}$ equals 1 if the municipality M is located within the maximum distance radius associated with the type of access point AP, and 0 otherwise,
- $w_{AP,ML}$ equals 1 if the municipality is located on one of the bus lines L connected to AP, (or one connection away in the mountains), 0 otherwise,
- $freq_L$ is the weighting factor associated with the level of servicing of line L.
