# Supplementary material for: Benefits of crowd-sourced GPS information for modelling the recreation ecosystem service
Source: PLoS One. 2018 Oct 15;13(10):e0202645. doi: 10.1371/journal.pone.0202645 (PMC6188625; doi:10.1371/journal.pone.0202645)
Supplement: S6 Appendix — (PDF) [file pone.0202645.s006.pdf]

S6 Appendix. Proximity of natural landscapes and waterscapes: quantitative details.

Added landscape attractiveness due to the proximity of waterscapes or natural land covers was set to 100% in their immediate vicinity and decreasing linearly with increasing distance, with a maximum impact distance set at d_* = 100m, beyond which it remained equal to 0. All proximity factors were then calculated using the same equation:

$$p(x) = 1 - \frac{d(x)}{d_*} \qquad \text{(S7.1)}$$

where:

- p(x) was the value of the proximity factor at pixel x
- d(x) the distance from x to the nearest pixel identified as water or a natural land cover.
