# Supplementary material for: Benefits of crowd-sourced GPS information for modelling the recreation ecosystem service
Source: PLoS One. 2018 Oct 15;13(10):e0202645. doi: 10.1371/journal.pone.0202645 (PMC6188625; doi:10.1371/journal.pone.0202645)
Supplement: S7 Appendix — (PDF) [file pone.0202645.s007.pdf]

S7 Appendix. Protection statuses used to compute the 'Conservation factor'.

Natural assets of the *Rhône-Alpes* region can be described using five types of areas:

- areas benefitting from a legal protection (table S8A, first rows)
- non-protected areas which are part of one or several natural inventories or zonings of acknowledged importance (table S8A, second rows)
- areas selected for the Regional Ecological Coherence Scheme (SRCE) which is not a protection status *per se* but must be taken into account in land planning (table S8A, third rows)
- non-urban remaining areas;
- urban areas.

We used these categories in this order as indicators of decreasing naturalness, ranging from 4 to 0.

Table S8A: list of natural protection statuses and inventories which were used to assess the level of conservation of the area. *ZNIEFF: zone of floristic, faunal and ecological value*

Legislation-protected areas	Central area of national parks (none in our case)<br>Prefectural order of biotope protection<br>National natural reserves<br>Forest biological reserves<br>Forests managed by the National Forest Office<br>Sensitive Natural Areas
Areas of acknowledged importance	Type 1 ZNIEFF* (species-oriented)<br>Type 2 ZNIEFF* (habitat-oriented)<br>Special Protection Areas for birds (SPAs, ZICO)<br>Regional Natural Park<br>Natura 2000 network (Birds Directive)<br>Natura 2000 network (Habitat Directive)<br>Sites inscrits<br>Peatland sites
RECS (SRCE)	Biodiversity reservoirs<br>Biological corridors<br>Water streams<br>Water bodies<br>Wetlands
