# Supplementary material for: Benefits of crowd-sourced GPS information for modelling the recreation ecosystem service
Source: PLoS One. 2018 Oct 15;13(10):e0202645. doi: 10.1371/journal.pone.0202645 (PMC6188625; doi:10.1371/journal.pone.0202645)
Supplement: S8 Appendix — (PDF) [file pone.0202645.s008.pdf]

S8 Appendix. Calculation of the scenic beauty factor (mountain only)

A viewshed analysis maps the area visible from a given point considering elevation and proximity of high vertical features such as forests and/or buildings. We computed individual viewsheds for each point at a 500m x 500m resolution grid covering the whole mountain territories in the study area and using a 50 x 50m resolution Digital Elevation Model (http://www.ign.fr/). We set visibility from urban and forested areas as well as orchards to zero, and elevation was increased by 25 meters in forested areas, 10m in urban areas and orchards, and 1m in vineyards.

For each point-related viewshed we considered 3 distance classes: the near zone lies within a 1.5km radius area around the point, the medium zone between 1.5 and 10km and the far zone between 10 and 50km. For each of these zones we calculated the perceived landscape beauty using a modified version of the linear regression model developed by Schirpke et al. [1]. These authors showed that perceived beauty in a northern alpine landscape can be predicted from landscape metrics through a regression model. From the original model, we used the two most important metrics only, namely the median shape index distribution and the modified Simpson's evenness index, since they each had the highest loadings in the original PCA model and were highly correlated to other similar indices (personal communication of the authors).

We calculated these two landscape metrics using Fragstats [2] over the ESNET Land Cover at a 15m x 15m resolution in the near zone, and over a composite land cover map of both the ESNET Land Cover and the CORINE Land Cover for the middle and far zones - as the viewshed from the points within the ESNET territory expands beyond the area covered by the ESNET Land Cover map. Following Schirpke et al.(1) an aggregation of land cover types was also carried out as differences between land cover types are less visible from afar, and re-aggregated the resulting map at 100m x 100m and 1km x 1km resolutions for the middle and far zones respectively.

Scenic beauty was then calculated for each distance zone around each point using the equation developed by Schirpke et al.(1); the total scenic beauty perceived from each point of view in the territory was then calculated as the weighted sum of scenic beauties for each distance zone of that point, using the weights also determined by Schirpke et al. [1].

$$SB_x = -0.378 + 6.601 * SHAPE.MD + 4.034 * MSIEI \qquad (S9.1)$$

and

$$SB_{tot} = 0.5 * SB_n + 0.3 * SB_m + 0.2 * SB_f \qquad (S9.2)$$

where:

- SB_{tot} the total scenic beauty for a panorama observed from a particular location
- SB_x the contribution of a distance zone x to the total scenic beauty of the panorama
- x being n, m, or f for the near, middle and far distance zones respectively
- $SHAPE.MD$ the median shape distribution index
- $MSIEI$ the modified Simpson's evenness intex

We did not include the scenic beauty factor in the lowland submodel. First, it was designed for assessing quality of mountain landscapes only, which was not suitable for the northwestern forested hills and agricultural plains of the study area. Secondly, the major scenic elements visible from lower areas are the cliffs of Chartreuse and Vercors, vertical features whose shapes are not captured by the 'aerial view' of a land cover map.

References

1. Schirpke U, Tasser E, Tappeiner U. Predicting scenic beauty of mountain regions. Landsc Urban Plan. 2013;111:1–12.

2. McGarigal K, Cushman SA, Neel MC, Ene E. FRAGSTATS: spatial pattern analysis program for categorical maps. 2002;
