# Supplementary material for: Benefits of crowd-sourced GPS information for modelling the recreation ecosystem service
Source: PLoS One. 2018 Oct 15;13(10):e0202645. doi: 10.1371/journal.pone.0202645 (PMC6188625; doi:10.1371/journal.pone.0202645)

S9 Appendix. Overview of the online validation survey.

General profile

Where do you live? - DEPARTEMENT - ▾ - COMMUNE - ▾

What is your main professional activity? (from French Classification of Occupations)

- ○ Farmer
- ○ Craft or trade worker, entrepreneur
- ○ Business manager
- ○ Employee, office clerk
- ○ Plant or machine operator, assembler
- ○ Intermediate profession
- ○ Retired
- ○ Student
- ○ Other, without any professional activity

What is your age category?

- AGE - ▾

Recreation profile

Which outdoor sports do you practice? (Multiple choices accepted)

- ☐ Mountaineering
- ☐ Canyoning
- ☐ Cycling
- ☐ Climbing
- ☐ Jogging
- ☐ Ski touring
- ☐ Snowshoe hiking
- ☐ Horse riding (outdoor)
- ☐ Hiking (pedestrian)
- ☐ Caving
- ☐ Skiing (alpine, cross-country, nordic)
- ☐ Nautical sports
- ☐ Trail running
- ☐ Paragliding
- ☐ Mountain-biking
- ☐ Other (specify) ____

Which outdoor leisure activity do you practice? (Multiple choices accepted)

- ☐ Art (photography, painting, etc.)
- ☐ Swimming
- ☐ Hand-picking
- ☐ Détente au bord de l’eau
- ☐ Naturalism
- ☐ Fishing
- ☐ Strolling

What is your frequency of practice, all activities considered?

- ○ Three times a week or more
- ○ Once a week or more
- ○ Once a month or more
- ○ Once every three month or more

You practice mostly:

- ○ On your own
- ○ Within a club
- ○ With your partner
- ○ With family
- ○ With friends

Do your practices vary between week days and week-ends?

- ○ Yes
- ○ No

If positive, how? (multiple choices accepted)

- ☐ I practice different activities
- ☐ I stay close to home during week days and go farer during weekends
- ☐ I practice on my own during week days and with relatives during weekends

Your usual transportation mode:

- ○ On foot
- ○ Bike
- ○ Car
- ○ Public transportation

Nature perception

What do you value most during your leisure time?
Rank options from the most (1) to the least (6) important

	1	2	3	4	5	6
Sports practice	○	○	○	○	○	○
Change of air	○	○	○	○	○	○
Contact with nature	○	○	○	○	○	○
Touring the region	○	○	○	○	○	○
Calm	○	○	○	○	○	○
Other (specify) ____	○	○	○	○	○	○

What are your criteria to choose a trip destination?
Rank options from the most (1) to the least (7) important

	1	2	3	4	5	6	7
A particular landscape	○	○	○	○	○	○	○
Remoteness from urban and urban-associated nuisance	○	○	○	○	○	○	○
A nice panorama	○	○	○	○	○	○	○
A particular landscape or geological feature (e.g. waterfall)	○	○	○	○	○	○	○
A well-preserved nature, regardless the landscape	○	○	○	○	○	○	○
Proximity	○	○	○	○	○	○	○
Other (specify) ____	○	○	○	○	○	○	○

Picture credits (next page)

http://www.espacebelledonne.fr/
http://www.lametro.fr/
http://www.isere-tourisme.com/
http://www.futura-sciences.com/
http://www.landscape-photo.net/
http://www.altituderando.com/
https://www.isere.fr/ (document: *Les chemins du paysage : un outil de connaissance des territoires de l'Isère*)

What kind of landscape do you look for?

Urban and peri-urban ☐

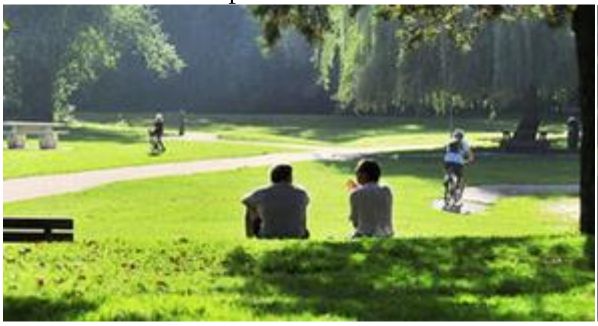

Mountain villages ☐

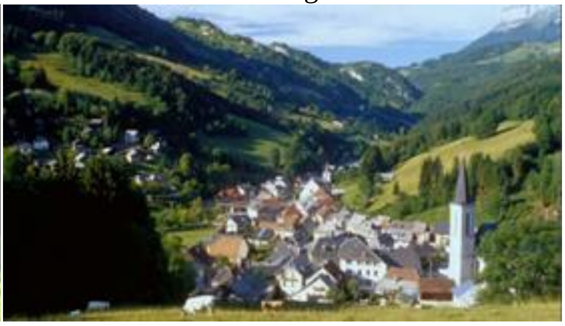

Agricultural mosaics ☐

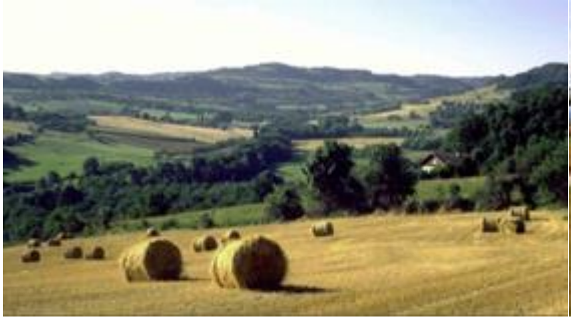

Openfield ☐

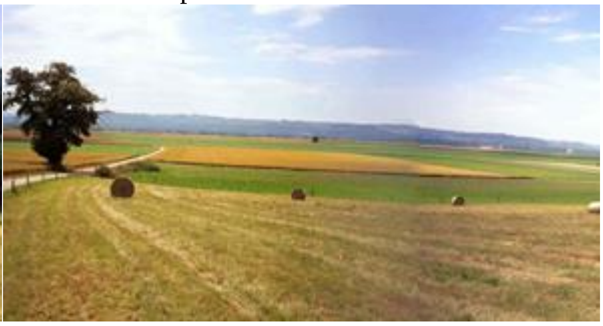

Broadleaved forest ☐

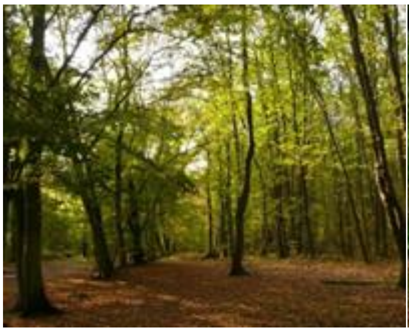

Needleleaved forest ☐

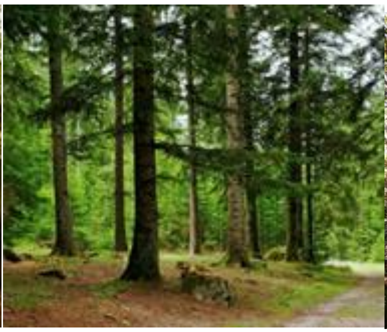

Mixed forests ☐

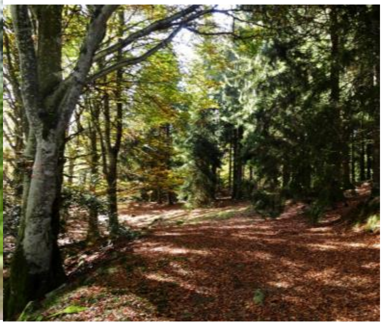

Grasslands ☐

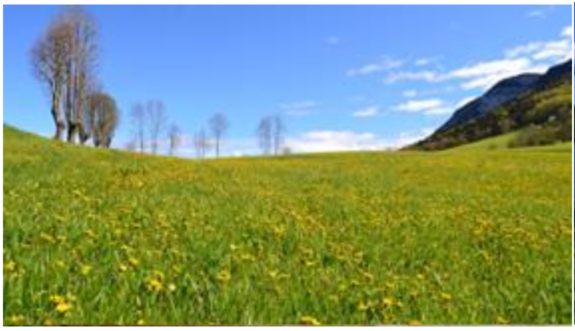

Waterscapes (lakes, swamps, rivers) ☐

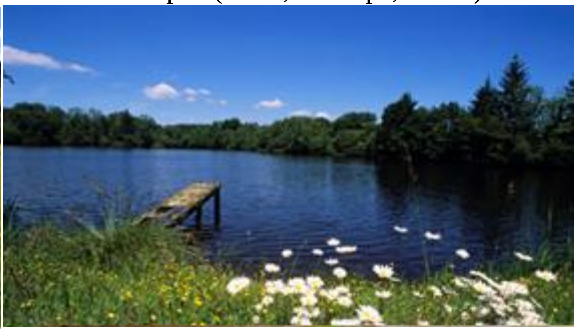

High mountain ☐

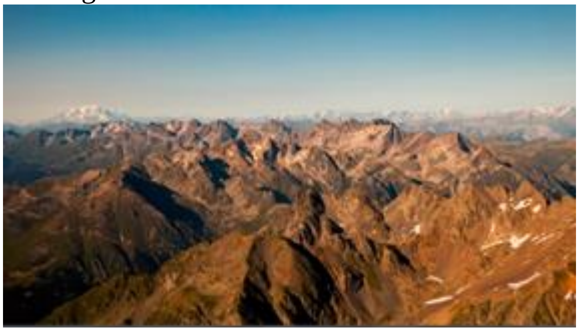

Lower mountain ☐

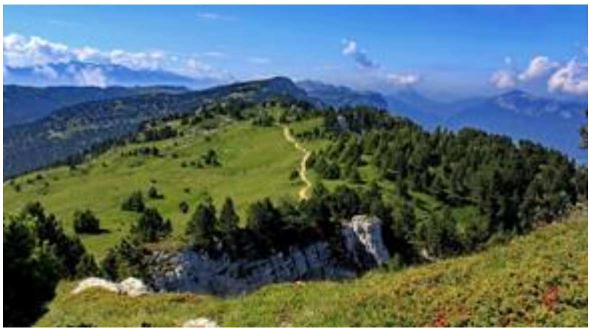

What are the main benefits you retrieve from your time out?

☐	Aesthetic:	enjoyment of the scenery
☐	Educational:	learnings derived from the observation and study of the surrounding nature and landscape
☐	Physical, psychological:	feeling of good physical and/or mental health
☐	Recreational:	pleasure derived from relaxation or any leisure activity
☐	Social:	pleasure derived from social exchange
☐	Spiritual:	sentiment religieux, de sacré, ou de cohérence avec l'univers
☐	Cultural:	'sense of place' associated with the presence of symbolic, traditional or historical elements
☐	Inspirational :	impressions likely to generate immediate or ulterior artistic creation

What factors could influence your intensity of practice?

		Increase	Decrease	Neutral
Evolution of touristic infra-structure	Creation of a leisure center (Center Parc Chambaran)	○	○	○
	Creation of a national park (PNR Belledonne, PNR Chambaran)	○	○	○
	Suppression of a ski resort (Sept Laux, Collet d'Allevard)	○	○	○
Major landscape change	Urban sprawl	○	○	○
	Forest extension	○	○	○
	Increase of agricultural mosaics	○	○	○
	Increased visitation	○	○	○
	Site degradation (pollution, lack of care)	○	○	○
Access	Restricted or paid access	○	○	○
	Decreased or suppressed public transpor-tation	○	○	○
	New road access to mountain ranges	○	○	○
	Increased public transportation (e.g. cable)	○	○	○
Other (specify)	▭			

Map

Please select districts you visit for outdoor leisure. Multiple choices are accepted.

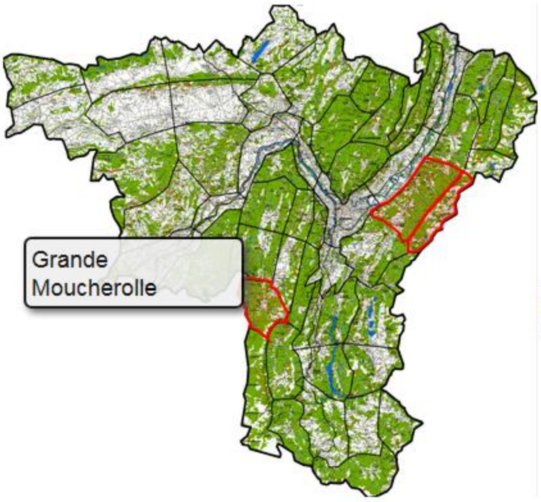

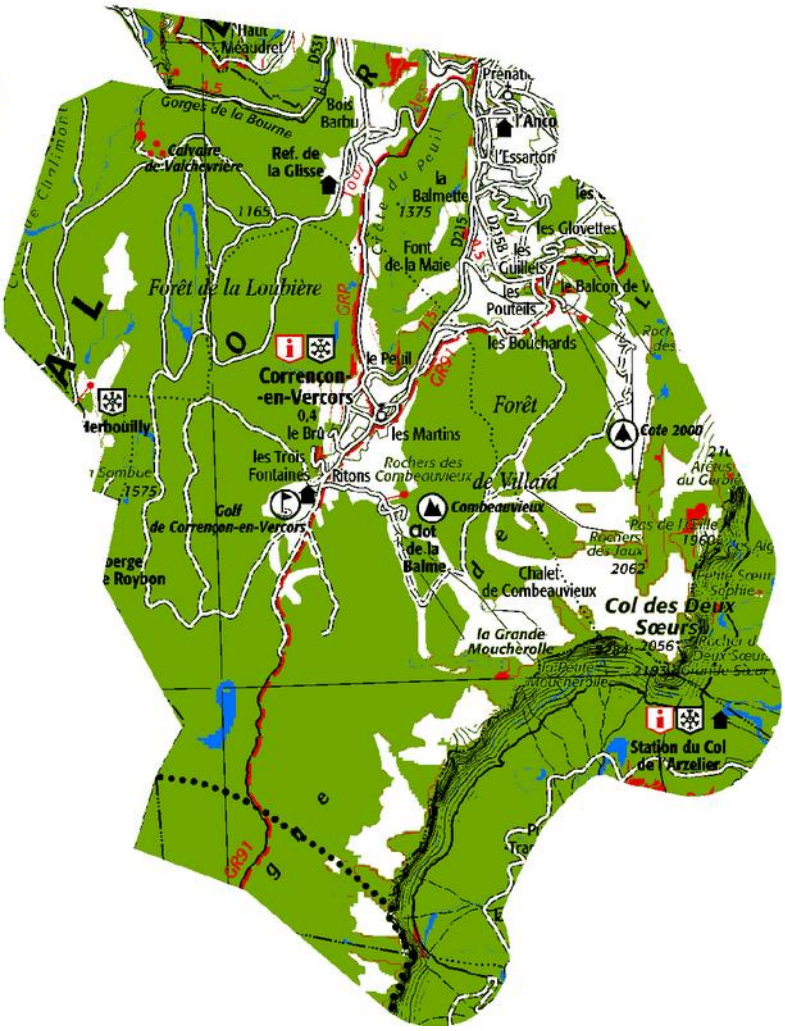

Supplement: S9 Appendix — (PDF) [file pone.0202645.s009.pdf]
