# Supplementary material for: Benefits of crowd-sourced GPS information for modelling the recreation ecosystem service
Source: PLoS One. 2018 Oct 15;13(10):e0202645. doi: 10.1371/journal.pone.0202645 (PMC6188625; doi:10.1371/journal.pone.0202645)
Supplement: S10 Appendix — (PDF) [file pone.0202645.s010.pdf]

S10 Appendix. Results from the online survey – Respondents' profiles and related figure.

A total of 986 people answered the survey. 51 people did not live in the Isère district or a neighbor district, and were ruled out of the analysis. 809 respondents lived within the study area. The spatial distribution of their geographic origins followed that of the population in general: 294 originated from the Grenoble city itself, 148 from the immediate neighbor cities; the remaining 493 respondents were mainly located in the valleys and mountain areas closest to Grenoble (Fig S8A).

Respondents were 38 years old on average (s.d.: 13 y.o.); the three most represented categories were managers (52%), employees (23%) and students (10%). They most likely belonged to two major socio-styles of the Grenoble area which are known as the 'Grenoble engineer' and the 'future Grenoble engineer'. Although unrepresentative of the whole population of the study area, they are completely representative of the fraction targeted by our model: wealthy and rather young adults as well as not so wealthy students but with more leisure time, who regularly spend time in mountain areas for recreational purposes (Fig S8B). Respondents had a high sport profile. They practiced a multiple sports: 3.8 sports on average (s.d. 2.8) out of the suggested list. The most popular sports were hiking, resort skiing, ski touring and climbing. Each of these four sports were practiced by more than 30% of respondents; 38% of respondents practiced at least 3 of them, and 67% at least 2 of them. Respondent were also regular sportsmen/women : 32% declared activities 3 times a week or more, and another 49% practiced at least once a week (Fig S8C).

Within the list of 15 suggested sports, 9 were practiced by at least 20% of the respondents, and 8 of them were directly taken into account in our model through the use of GPS tracks. In the same manner, the most popular non sportive leisure activities, strolling and bathing (practiced by 62 and 44% of respondents respectively) were also included in our model, although not mapped using GPS tracks (Fig S8C). This multiplicity of practices, alongside with the polyvalent profile of respondents, justified our multi-activity recreation model in this area, and confirmed the relevance of a multifunctional approach.

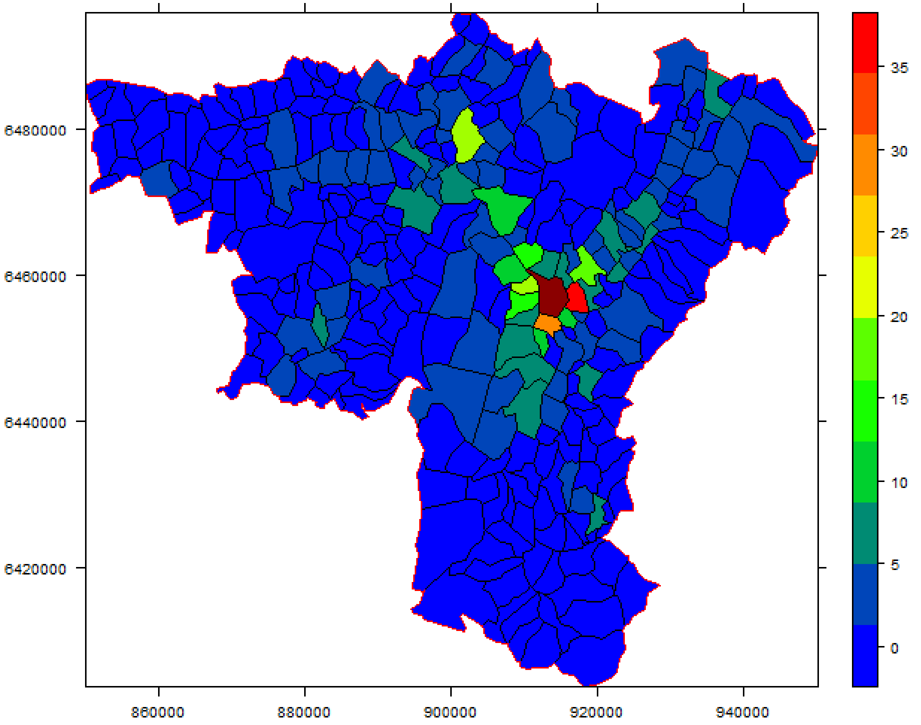

A. Geographical origin within the study area – number of respondents per municipality; Grenoble, an outlier, is plotted in brown (n=294).

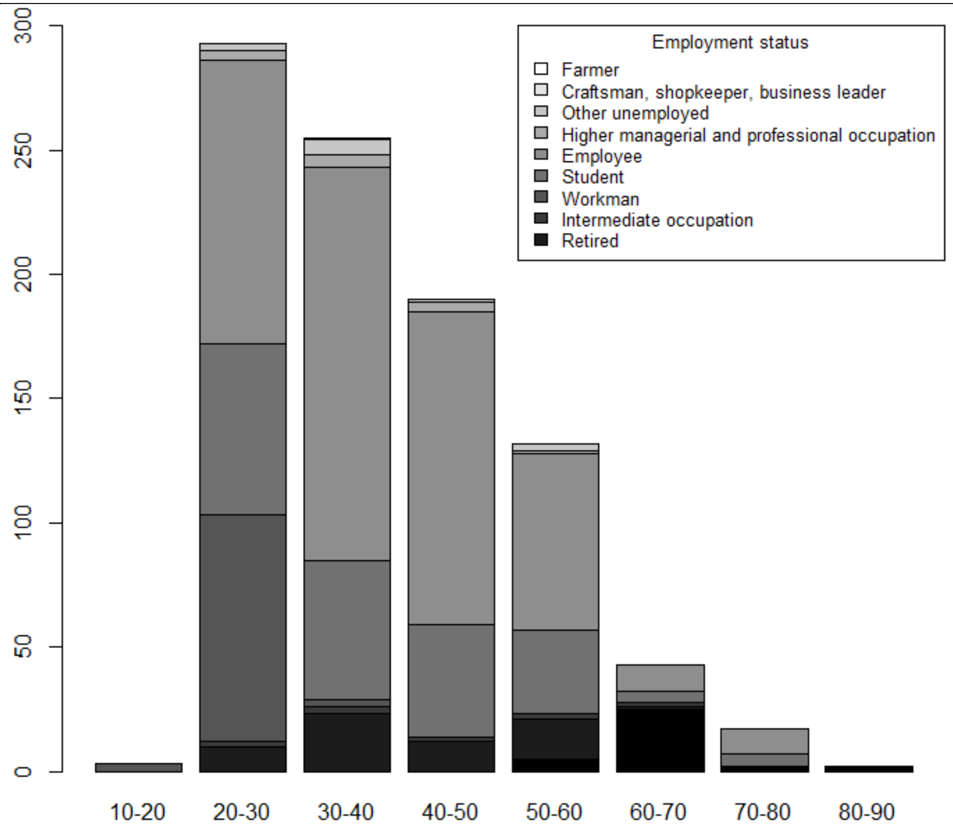

B. Age category and employment status. The most represented job status are: Managers, Employees, and Students for the 20-30 y.o., and Retired for the 60-70 y.o..

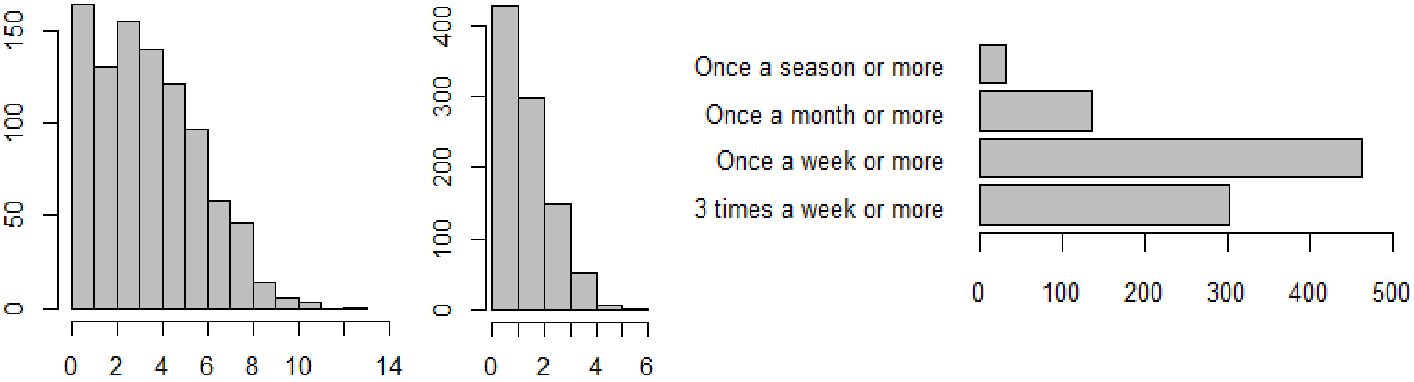

C. Recreational profiles.

From left to right :

Number of sport practiced per person (y-axis: number of people; x-axis: number of sports)

Number of non-sport leisure activities practiced per person (y-axis: number of people; x-axis: number of activities)

Practice frequences (x-axis: number of people; y-axis: frequence category)
