# Supplementary material for: Benefits of crowd-sourced GPS information for modelling the recreation ecosystem service
Source: PLoS One. 2018 Oct 15;13(10):e0202645. doi: 10.1371/journal.pone.0202645 (PMC6188625; doi:10.1371/journal.pone.0202645)
Supplement: S1 Table — (PDF) [file pone.0202645.s011.pdf]

S1 Table. Number of GPS tracks or recreation sites per activities.

Asterisk-marked activities were not modelled with GPS tracks.

**Activity**	**n**
Mountain biking	1561
Hiking	1035
Skiing	748
Trail running	483
Cycling	260
Horse riding	230
Climbing sites*	157
Snowshoe hiking	40
Ice climbing	28
Mountaineering	36
Multi-pitch climbing	30
Lake leisure areas*	13
