# Supplementary figures and images for: Benefits of crowd-sourced GPS information for modelling the recreation ecosystem service
Source: PLoS One. 2018 Oct 15;13(10):e0202645. doi: 10.1371/journal.pone.0202645 (PMC6188625; doi:10.1371/journal.pone.0202645)

**S1 Fig. Intermediate factor maps.** Standardized Values range from 0 (white) to 1 (black).

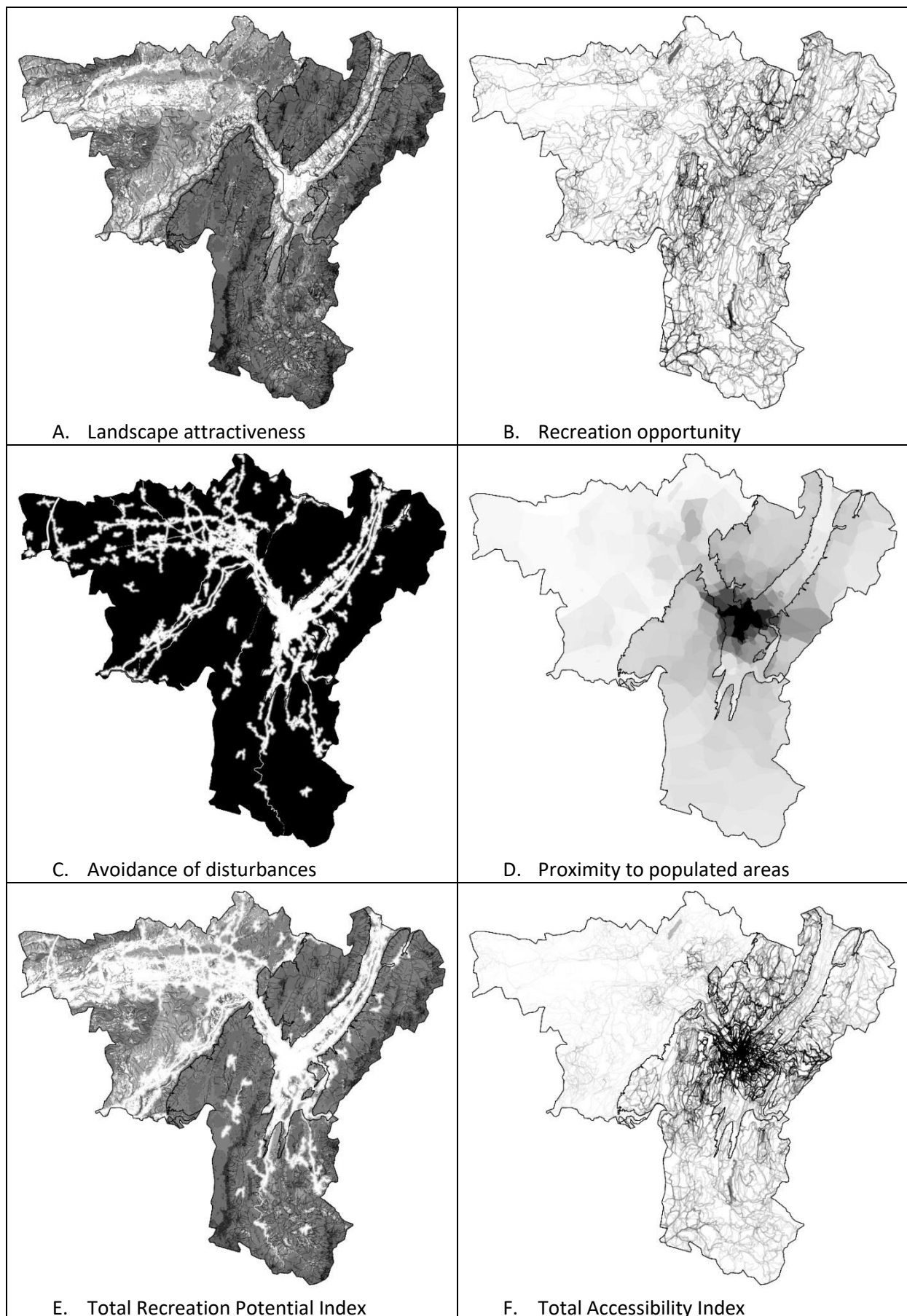

Supplement: S1 Fig — (PDF) [file pone.0202645.s012.pdf]

**S4 Fig. Results from the online survey - Service perception (A,C) and factor preferences (B,D)**

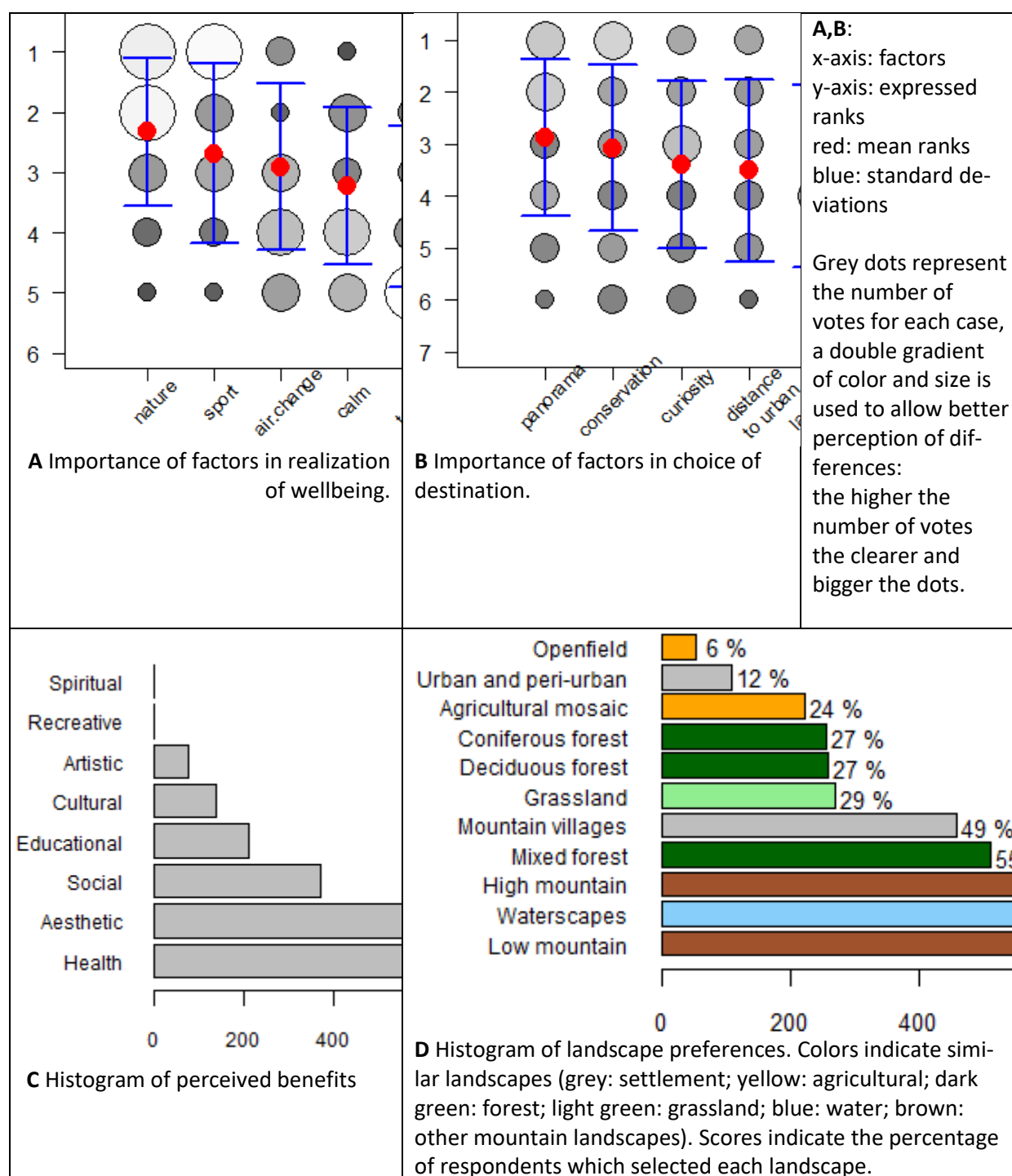

Supplement: S4 Fig — (PDF) [file pone.0202645.s015.pdf]
