# Supplementary material for: Benefits of crowd-sourced GPS information for modelling the recreation ecosystem service
Source: PLoS One. 2018 Oct 15;13(10):e0202645. doi: 10.1371/journal.pone.0202645 (PMC6188625; doi:10.1371/journal.pone.0202645)

**S2 Fig. Spatial extents of opportunity networks.** These networks were produced by pooling all GPS tracks downloaded from crowd-sourced sport-oriented websites. The total number of GPS tracks used for each sport can be found in S3 Table.

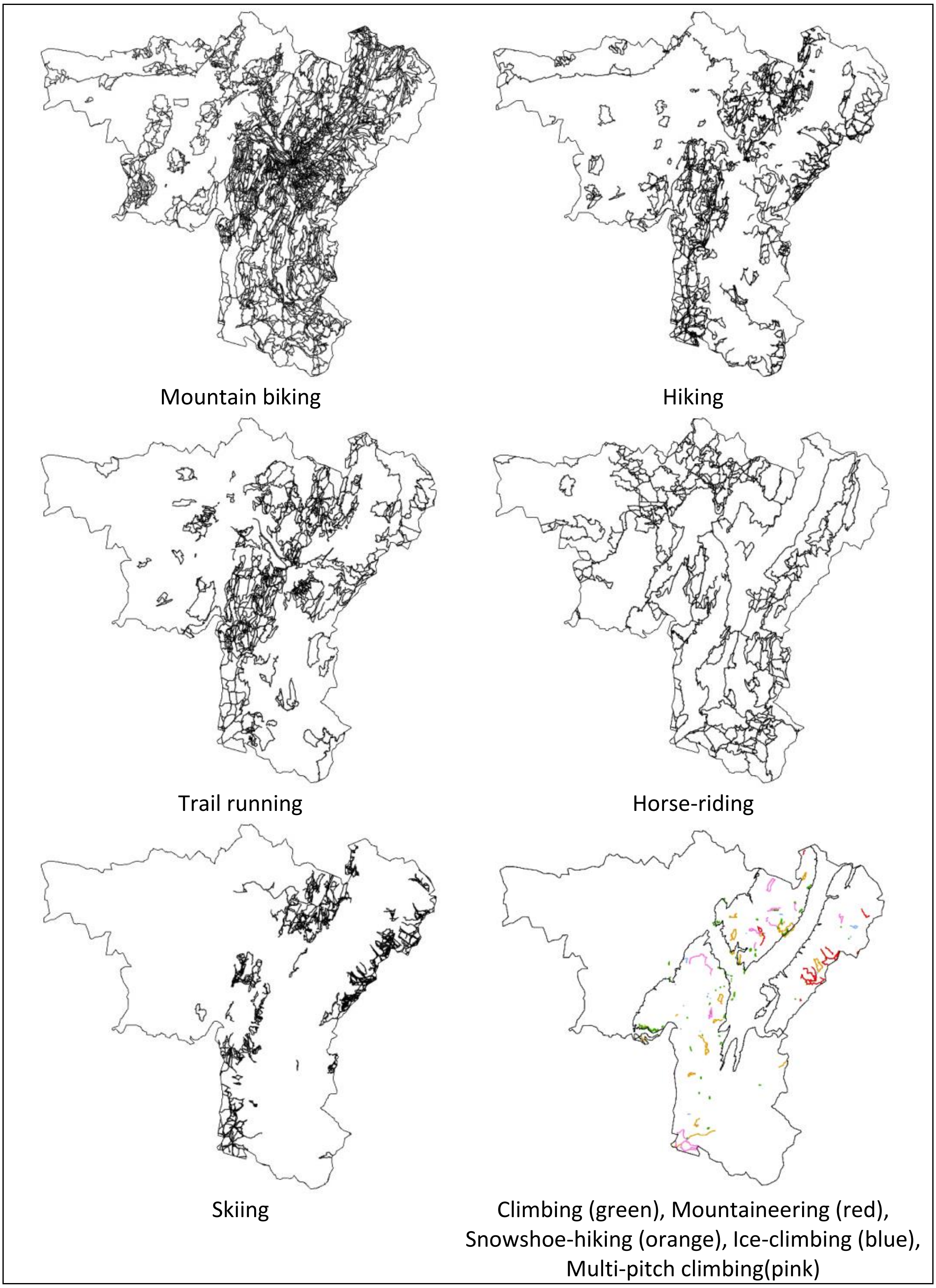

Supplement: S2 Fig — (PDF) [file pone.0202645.s013.pdf]
