# Supplementary material for: Benefits of crowd-sourced GPS information for modelling the recreation ecosystem service
Source: PLoS One. 2018 Oct 15;13(10):e0202645. doi: 10.1371/journal.pone.0202645 (PMC6188625; doi:10.1371/journal.pone.0202645)

S3 Fig. Visitation rates. The opportunity networks were resampled at a resolution of 250m x 250m. Values express the number of GPS tracks which were present per pixel. Color scales are equivalent in all four maps except that the maximum values differ; hiking: 145, skiing: 200, trail running: 97; mountain biking: 530.

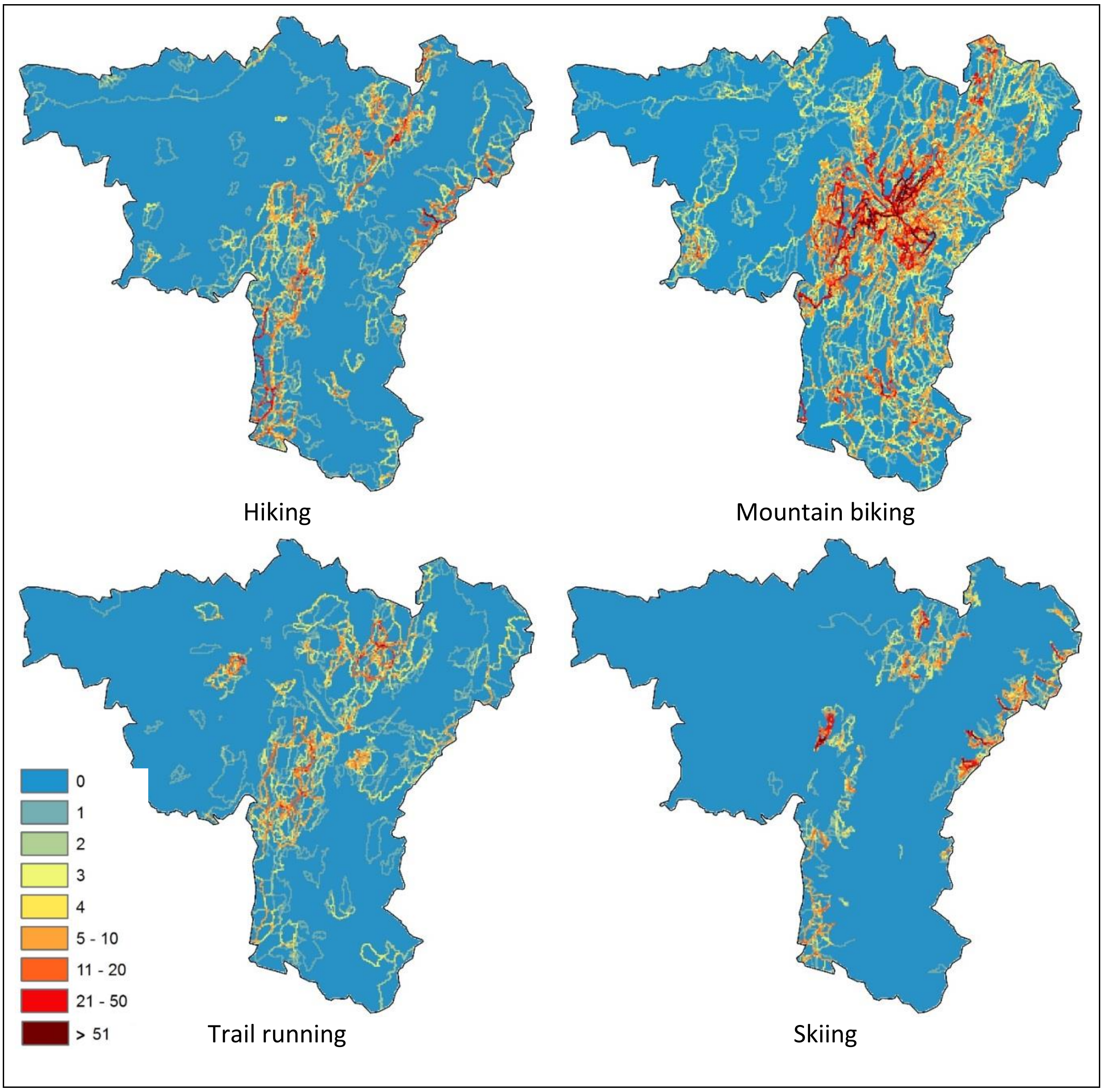

Supplement: S3 Fig — (PDF) [file pone.0202645.s014.pdf]
